# Impact of Withholding Breastfeeding at the Time of Vaccination on the Immunogenicity of Oral Rotavirus Vaccine—A Randomized Trial

**DOI:** 10.1371/journal.pone.0127622

**Published:** 2015-06-02

**Authors:** Asad Ali, Abdul Momin Kazi, Margaret M. Cortese, Jessica A. Fleming, SungSil Moon, Umesh D. Parashar, Baoming Jiang, Monica M. McNeal, Duncan Steele, Zulfiqar Bhutta, Anita K. M. Zaidi

**Affiliations:** 1 Department of Pediatrics and Child Health, Aga Khan University, Karachi, Pakistan; 2 Division of Viral Diseases, National Center for Immunization and Respiratory Diseases, Centers for Disease Control and Prevention, Atlanta, Georgia, United States of America; 3 Vaccine Access and Delivery, Program for Appropriate Technology in Health (PATH), Seattle, WA, United States of America; 4 Department of Pediatrics, Division of Infectious Diseases, Cincinnati Children’s Hospital Medical Center, Cincinnati, OH, United States of America; Public Health England, UNITED KINGDOM

## Abstract

**Background:**

Breast milk contains anti-rotavirus IgA antibodies and other innate immune factors that inhibit rotavirus replication *in vitro*. These factors could diminish the immunogenicity of oral rotavirus vaccines, particularly if breastfeeding occurs close to the time of vaccine administration.

**Methods:**

Between April 2011 and November 2012, we conducted an open label, randomized trial to compare the immunogenicity of Rotarix (RV1) in infants whose breastfeeding was withheld one hour before through one hour after vaccination with that in infants breastfed at the time of vaccination. The trial was conducted in the peri-urban area of Ibrahim Hyderi in Karachi, Pakistan. Both groups received three doses of RV1 at 6, 10 and 14 weeks of age. Seroconversion (anti-rotavirus IgA antibodies ≥20 U/mL in subjects seronegative at 6 weeks of age) following three vaccine doses (6, 10 and 14 weeks) was determined at 18 weeks of age (primary objective) and seroconversion following two doses (6 and 10 weeks) was determined at 14 weeks of age (secondary objective).

**Results:**

Four hundred eligible infants were randomly assigned in a 1:1 ratio between the withholding breastfeeding and immediate breastfeeding arms. Overall, 353 (88.3%) infants completed the study according to protocol; 181 in the withholding breastfeeding group and 172 in the immediate breastfeeding group. After three RV1 doses, anti-rotavirus IgA antibody seroconversion was 28.2% (95% CI: 22.1; 35.1) in the withholding arm and 37.8% (95% CI: 30.9; 45.2) in the immediate breastfeeding arm (difference: -9.6% [95% CI: -19.2; 0.2] p=0.07). After two doses of RV1, seroconversion was 16.6% (95% CI: 11.9; 22.7) in the withholding arm and 29.1% (95% CI: 22.8, 36.3) in the immediate breastfeeding arm (difference: -12.5% [95% CI: -21.2,-3.8] p=0.005).

**Conclusions:**

Withholding breastfeeding around the time of RV1 vaccine administration did not lead to increased anti-rotavirus IgA seroconversion compared with that seen with a breastfeed at the time of vaccination. On the contrary, IgA seroconversion in infants immediately breastfed tended to be higher than in those withheld from a feeding. Our findings suggest that breastfeeding should be continued adlib around the time of rotavirus vaccination and withholding breastfeeding at that time is unlikely to improve the vaccine immunogenicity.

**Trial Registration:**

ClinicalTrials.gov NCT01199874

## Introduction

Rotavirus is the leading cause of moderate to severe diarrhea in infants globally, causing an estimated 453,000 deaths in 2008 [1]. Two oral rotavirus vaccines, 2-dose Rotarix (GlaxoSmithKline Biologicals, Rixensart, Belgium) (RV1) and 3-dose RotaTeq (Merck and Co., Inc., Whitehouse Station, NJ, USA) (RV5) are in use in in many countries. While highly efficacious (~85%) against severe rotavirus disease in industrialized countries, these vaccines show substantially lower immunogenicity and efficacy (~51%-66%) against severe rotavirus in lower income countries where most deaths from rotavirus occur [2, 3]. Factors that could explain this difference in vaccine performance are increasingly becoming the focus of attention, given the potential for significant impact by even modestly improving the effectiveness of rotavirus vaccines in developing countries.

Breastfeeding has been postulated as one possible factor that might impair rotavirus vaccine performance [4]. Breast milk contains both secretory anti-rotavirus IgA antibodies as well as innate immune factors such as lactoferrin, lactoadherin, mucin and butyrophilin which are inhibitors of rotavirus replication [4]. In an *in vitro* study in which breast milk from Bangladeshi mothers was mixed with an equal volume of live serotype 1 rotavirus Wa strain, about 30% and 60% of breast milk samples neutralized 99% and 90% of the virus, respectively [5]. Recent studies by Moon *et al* demonstrated that the titers of neutralizing activity (NA) against three rotavirus vaccine strains, RV1, RV5 G1 and 116E were higher in the breast milk of mothers in India compared with mothers in the United States [6, 7]. This regional variation in NA could contribute to the difference in immunogenicity of RV vaccine in developed and developing countries if indeed infants are receiving vaccine at the time that breast milk is also present in sufficient quantity to reduce the effective inoculum.

If the high levels of NA and other factors in breast milk reduce the immunogenicity of the vaccine when breast milk is provided at the same time as RV vaccine, withholding breastfeeding for some time before and after vaccine administration could increase vaccine immunogenicity. While withholding a feed may be difficult to implement programmatically, it is important to understand if breast milk is an important factor contributing to the reduced performance of current live oral rotavirus vaccines in developing countries so that programmatic methods and/or new vaccines may be designed to overcome the effect. We therefore conducted a randomized trial in Pakistan (where virtually all mothers breastfeed their young infants), to compare the immunogenicity of RV1 in infants whose breastfeeding was withheld one hour before through one hour after vaccination with each RV1 dose withthat in infants who were breastfed at the time of vaccination. We also evaluated the effect of rotavirus neutralizing activity in breast milk on the immunogenicity of RV1.

## Methods

### Participants

The study was conducted in the peri-urban area of Ibrahim Hyderi in Karachi, Pakistan, where the Department of Pediatrics and Child Health of Aga Khan University has conducted demographic surveillance, including pregnancy and newborn surveillance, since 2008. It is a low-income community of predominantly fishermen. Neonatal and under 5 year mortality in this population were 40.7 and 78.7 per 1000 live births in 2012, respectively.

Study recruitment was done from 11 April 2011 to 25 Jun 2012. Parents were informed of the study when their infants were four weeks of age, and if interested, written informed consent was obtained and their children enrolled at six weeks of age. Only infants whose mothers intended to breastfeed their babies for at least 18 weeks were included in the trial. Infants were excluded if they had a birth weight less than 1500 grams, received rotavirus vaccine outside of the study, had received any immunoglobulin or blood products since birth, were on immunosuppressive drugs, or had used any investigational drug or vaccine within 30 days of the first dose of study vaccine. Final follow up of the last participant was completed on 24 Sep 2012. The trial was approved by the institutional review boards of Aga Khan University, the United States Centers for Disease Control and Prevention, and the Western Institutional Review Board (Olympia, WA, USA). The study was conducted in accordance with the principles of the Declaration of Helsinki and in compliance with good clinical practice guidelines; ClinicalTrials.gov registration number NCT01199874.

### Randomization and masking

This randomized trial of 400 children included two intervention arms with treatment allocation of 1:1. Infants were randomized to one of two groups, according to computer-generated randomization codes provided in sequentially numbered and assigned envelopes. The study was partially blinded for logistical reasons; both participants and investigators were aware of the group assignment but the laboratory scientists testing for rotavirus antibodies were not.

### Procedures

Both study groups received three doses of the lyophilized formulation of RV1 at 6, 10 and 14 weeks of age. The regimen allowed for the assessment of the impact of breastfeeding in both the conventional two dose schedule as well as the three dose regimen which was being studied in a parallel trial [8]. Mothers randomized to the withholding breastfeeding arm were instructed to withhold breastfeeding for one hour before and after each RV1 dose at the study clinic; mothers were allowed to breastfeed the infant in the clinic, if they desired, before the monitored withholding period began. At the end of the withholding period (one hour after RV1 receipt), mothers often left the clinic before resuming feeding so the exact duration of withholding beyond the second hour was not consistently available. In the immediate breastfeeding group, mothers were asked to breastfeed their baby for at least 20 minutes at the study clinic, and the child was administered RV1 as soon as possible after the completion of breastfeeding (maximum, 10 minutes after). The vaccination visits for the two arms occurred on different days of the week to ensure the appropriate feeding for each infant. The timing and minimum duration of the withholding time or breastfeeding and the timing of the RV1 dose were monitored closely and the success and timing of each step was documented by a study nurse. At each vaccine visit, breast milk samples were expressed by both groups of mothers before the dose was administered. RV1 was provided along with trivalent oral poliovirus vaccine (tOPV) and pentavalent vaccine (diphtheria, tetanus, whole cell pertussis, hepatitis B and *Haemophilus influenzae* type b), unless either had been administered earlier at another facility. Vaccinations were postponed if the child was suffering from fever ≥38.0°C, had vomited during each of the last three feedings or met WHO criteria for some or severe dehydration [9]. The study schedule is summarized in Table 1.

**Table 1:** Vaccination and Specimen Collection Schedule for both Group 1 and Group 2.

Study Visit 1 (V1)	Study Visit 2 (V2)	Study Visit 3 (V3)	Study Visit 4 (V4)
6 weeks	10 weeks	14 weeks	18 weeks
Age: 42 to 55 days	Age: 25 to 41 days post V1	Age: 25 to 41 days post V2	Age: 25 to 41 days post V3
Blood draw		Blood draw	Blood draw
Breast milk sample	Breast milk sample	Breast milk sample	
RV1 and EPI vaccinations	RV1 and EPI vaccinations	RV1 and EPI vaccinations	
Safety evaluation	Safety evaluation	Safety evaluation	

### Laboratory Assays

Anti-rotavirus IgA antibody was measured in serum collected at age 6 weeks (pre-vaccination), 14 weeks and 18 weeks in all infants. The concentration of anti-rotavirus IgA antibody in serum was measured using an enzyme-linked immunosorbent assay (ELISA) previously described and expressed as Units per mL (U/mL)[10]. Neutralizing antibodies, presumed to be maternally derived (MDNA), were measured in the infants' serum collected at 6 weeks using a neutralization assay against rotavirus strain 89–12, the precursor to the RV1 vaccine strain, following methods previously described and expressed as the titer of the serum which represents 60% neutralization of the virus [11].

Anti-rotavirus IgA was measured in breast milk samples collected at 6, 10 and 14 weeks using an ELISA previously described and expressed as titers [6]. Rotavirus neutralizing activity in breast milk (BMNA) was assessed in the sample collected at 10 weeks using a neutralization assay against the vaccine strain (RV1), following methods previously described [6]. Neutralizing titer in breast milk was determined as the reciprocal of the highest dilution that showed greater than 70% reduction in the absorbance value compared with that in the virus-only control. The IgA and neutralizing assays used for the serum specimens were different from those used for the breastmilk specimens and hence titers cannot be directly compared.

### Safety Assessment

All infants were observed for at least 30 minutes after vaccine administration and parents were asked to seek care at the study clinic for any illnesses in participants during the study period. All serious adverse events (SAEs) were reported to an independent safety monitor who characterized possible relationship to study product.

### Outcomes

Seroconversion was defined as a serum concentration of anti-rotavirus IgA antibodies ≥20 U/mL in subjects who were seronegative (IgA <20 U/mL) at 6 weeks of age. Seroconversion following three doses (6, 10 and 14 weeks) was determined at 18 weeks of age (primary objective) and seroconversion following two doses (6 and 10 weeks) was determined at 14 weeks of age (secondary objective).

Analyses were conducted on the per protocol cohort, which included infants meeting all inclusion and no exclusion criteria, who were anti-rotavirus IgA seronegative at 6 weeks, received RV1 vaccines and blood draws according to schedule with no significant deviations, and remained in the study through 18 weeks. The breastfeeding requirements for the per-protocol cohort included documentation that the observed pre-dose and post-dose withholding period were each ≥ 60 minutes (withholding arm) or infants received RV1 within 10 minutes after an observed successful breastfeed (immediate arm).

We expected that any independent role of BMNA in impacting seroconversion would be most likely revealed by examining infants in the immediate feeding arm with low MDNA. We chose *a priori* to measure BMNA in the 10-week breast milk sample for two reasons: 1) it is expected that the 10-week vaccine dose is less impacted by serum MDNA compared to the 6-week dose, thus the independent effect of BMNA would be better assessed with a sample collected at a later dose, and 2) many infants would likely seroconvert after two vaccine doses and hence evaluating BMNA around the third dose (14 weeks) would be less informative. Therefore, infants in the immediate arm were divided into a high and low BMNA groups using the median BMNA titer as the cut point, and similarly were also divided into high and low MDNA groups using their median MDNA titer, and comparisons were made of serconversion following RV1 administration in these sub-groups.

### Statistical analysis

Serum rotavirus IgA seroconversion between the withholding and the immediate breastfeeding arms were compared using the Fisher Exact test and 95% confidence intervals (CI) of the differences in seroconversion were calculated based on the Newcombe-Wilson method without continuity correction. Serum anti-rotavirus IgA geometric mean titers (GMT) were compared across arms using a t- test for all infants as well as seroconverted infants; t-test was used to compare the log-transformed titers and 95% CIs were constructed based on the t-test. GMTs and 95% CI of GMTs were results of anti log-transformation of the log-transformed results. A value of 10 U/mL was assigned to infants with a IgA level of <20 U/mL for statistical purposes. To assess the overall relationship between MDNA in the 6 week serum sample and BMNA in the 10 week sample, Spearman’s rank correlation was used for both arms combined.

Enrollment of 200 infants in each arm was estimated to provide 85% power to detect a 17.5% difference in seroconversion after three vaccine doses between the withholding arm and the immediate breastfeeding arm, using a 2-sided Chi Square test (alpha 0.05), assuming a 45% seroconversion in the immediate breastfeeding arm, 5% IgA seropositivity at 6 weeks, and a 20% drop out during the study. Data analyses were performed using SAS and Stata software.

### Role of Funding Source

The study was supported by PATH through funding from the Global Alliance for Vaccines and Immunization (GAVI). The corresponding author had full access to all the data and had final responsibility for the decision to submit for publication.

## Results

### Study Population

**Fig 1:**
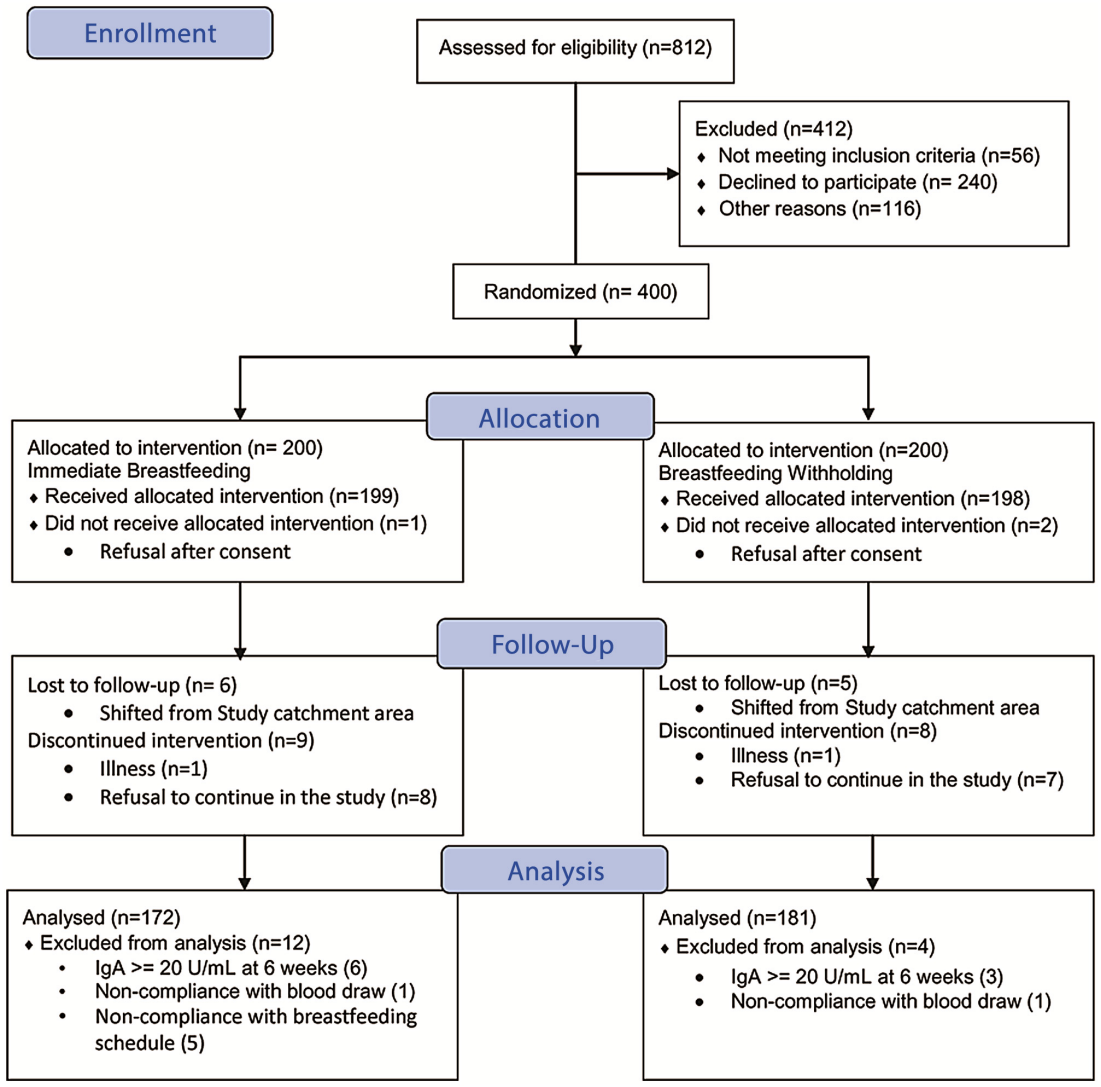
Study Assignment and Follow-Up of Participants.

Study arms were similar with respect to age, gender, length, weight, and family/household characteristics at the time of enrollment (Table 2). Of the 400 children enrolled, nine (2.2%) were anti-rotavirus IgA positive in serum collected at age 6 weeks, and were therefore dropped. Overall, 353 (88.3%) infants completed the study according to protocol (Fig 1).

**Table 2:** Baseline Characteristics and Age at Study Visit.

	Withholding breastfeeding Arm(N = 200)		Immediate breastfeeding Arm (N = 200)	
	N	% or Mean (SD)	n	% or Mean (SD)
Male; %	89	45.0	108	54.0
Age in Weeks; mean (SD)	200	6.3 (0.5)	200	6.3 (0.5)
Length (cm); mean (SD)	200	53.5 (2.5)	200	53.7 (2.5)
Weight (g) at 6 weeks; mean (SD)	200	3939.1 (619.2)	200	4047.5 (594.7)
FOC (cm) at 6 weeks; mean (SD)	200	36.7 (1.6)	200	36.9 (2.0)
Age of Mother (years); mean (SD)	200	26.1 (4.5)	200	25.6 (5.1)
Mother Years of Schooling; mean (SD)	200	4.7 (4.5)	200	4.7 (4.5)
Father Years of Schooling; mean (SD)	200	5.5 (4.7)	200	5.6 (4.7)
Type of milk				
Exclusive breastfed;%	153	76.5	158	79.0
Breast milk plus formula;%	46	23.0	42	21.0
Exclusive formula milk;%	1	0.5	-	-
Main source of water				
Piped in/out dwelling; %	106	53.0	115	57.5
Tube/deep tube well; %	1	0.5	1	0.5
Surface well; %	-	-	1	0.5
Surface water; %	33	16.5	26	13.0
Bottled/filtered; %	50	25.0	49	24.5
Other; %	10	5.0	8	4.0
Main material of roof				
Jute/bamboo/mud; %	4	2.0	4	2.0
Tin; %	10	5.0	6	3.0
Cement/concrete; %	186	93.0	189	94.5
Other; %	-	-	1	0.5
Main type of toilet				
Septic tank/modern toilet; %	14	7.0	17	8.5
Pit latrine, water sealed; %	25	12.5	28	14.0
Pit latrine, not water sealed; %	161	80.5	155	77.5
Place of delivery				
Home; %	75	37.5	70	35.0
Hospital/Clinic; %	125	62.5	130	65.0
Age of visit in weeks^†^; mean (SD)				
Visit 2	181	10.4 (0.5)	172	10.4 (0.6)
Visit 3	181	14.6 (0.6)	172	14.6 (0.6)
Visit 4	181	18.7 (0.7)	172	18.7 (0.7)

^†^per-protocol population

In the per-protocol population, the mean age of infants at each study visit was similar across intervention arms. tOPV vaccine was given concomitantly with each dose of RV1 in ≥ 95% of participants in each arm; the remaining children had documentation of receiving tOPV doses from an outside facility. Birth dose of tOPV had been received, per parental report, by 87% of infants (similar in both arms), and additional monovalent OPV or bivalent OPV doses were received by study infants during vaccine campaigns that occurred during the trial period. The GMT of MDNA against rotavirus, measured in infants’ serum at age 6 weeks, was similar in the withholding and immediate breastfeeding arms; 116.2 (95%CI: 99.1; 136.2) and 96.5 (95%CI: 81.4;114.3), respectively.

### Immune Response to RV1

After three RV1 doses administered at 6, 10, and 14 weeks, anti-rotavirus IgA antibody seroconversion was 28.2% (95% CI: 22.1; 35.1) in the withholding arm and 37.8% (95% CI: 30.9; 45.2) in the immediate arm (difference: -9.6% [95% CI: -19.2; 0.2] p = 0.07) (Table 3). Anti-rotavirus IgA GMT at 18 weeks was 20.4 U/mL and 23.1 U/mL in the withholding and immediate breastfeeding arm, respectively; the GMT of the seropositive infants were not significantly different between arms (Table 4).

**Table 3:** Percent Anti-Rotavirus IgA Seroconversion.

Sample	Withholding breastfeeding Arm	Withholding breastfeeding Arm	Withholding breastfeeding Arm	Immediate breastfeeding Arm	Immediate breastfeeding Arm	Immediate breastfeeding Arm		
	(N = 181)	(N = 181)	(N = 181)	(N = 172)	(N = 172)	(N = 172)	Difference in seroconversion	*P* value
	n	%	95% CI	N	%	95% CI		
6 week	0	0		0	0			
14 week	30	16.6	(11.9; 22.7)	50	29.1	(22.8; 36.3)	-12.5 (-21.2; -3.8)	0.005
18 week	51	28.2	(22.1; 35.1)	65	37.8	(30.9; 45.2)	-9.6 (-19.2, 0.2)	0.07

*Post hoc comparison for seroconversion at 14 or 18 weeks of age: Withholding (n = 56, 30.9%); Immediate (n = 76, 44.2%); difference -13.2 (95% CI -23.3; -3.2), p = 0.011

**Table 4:** Anti-Rotavirus IgA Geometric Mean Titers (GMT).

	Withholding Breastfeeding arm			Immediate Breastfeeding arm			
Sample	n	GMT (U/mL)	95% CI	N	GMT (U/mL)	95% CI	P value
All subjects							
6 week	181	10.0	(10.0; 10.0)	172	10.0	(10.0; 10.0)	
14 week	181	16.4	(13.7; 19.5)	172	19.8	(16.5; 23.7)	0.139
18 week	181	20.4	(16.9; 24.6)	172	23.1	(19.1; 28.0)	0.362
Seroconverted subjects^†^							
14 week ^‡^	30	194.4	(126.2; 299.3)	50	104.4	(77.3; 140.9)	0.016
18 week ^§^	51	125.4	(91.0; 172.9)	65	91.8	(69.1; 121.8)	0.147

^†^Subjects with anti-rotavirus IgA ≥20 U/mL at 14 or 18 weeks, respectively.

^‡^Includes only anti-rotavirus IgA seropositive subjects at 14 weeks

^§^Includes only anti-rotavirus IgA seropositive subjects at 18 weeks

After two doses of RV1 at 6 and 10 weeks, anti-rotavirus IgA antibody seroconversion was 16.6% (95% CI: 11.9; 22.7) in the withholding arm and 29.1% (95% CI: 22.8, 36.3) in the immediate breastfeeding arm (difference: -12.5% [95% CI: -21.2, -3.8] p = 0.005) (Table 3). Anti-rotavirus IgA GMT post-vaccination after the two dose regimen was 16.4 U/mL in the withholding arm (seroconverters and non-seroconverters included) and 19.8 U/mL in the immediate breastfeeding arm (p = 0.139) (Table 4). At age 14 weeks, the GMT of infants who were seropositive was significantly higher in the withholding arm compared to the immediate arm (194.4 U/mL (95%CI: 126.2; 299.3) vs.104.4 U/mL (95%CI: 77.3; 140.9) p = 0.016).

In the withholding arm, 17.2% (26/151) of those who were seronegative at 14 weeks were seropositive at 18 weeks following the third dose, and 16.7% (5/30) of those seropositive at 14 weeks were seronegative at 18 weeks (S1 Fig). In the immediate breastfeeding arm, 21.3% (26/122) of those seronegative at 14 weeks became seropositive following a third dose and 22.0% (11/50) of those seropositive became seronegative. Within both arms, the IgA GMT of those infants who had seroconverted by 14 weeks after 2 doses was similar to their GMT at 18 weeks, suggesting that the 3rd dose did not substantially boost the immune response in these infants (S1 Fig).

### Evaluation of Breast Milk Rotavirus Neutralizing Activity

Anti-rotavirus IgA titers in breast milk samples were similar between the two arms at each time point 6, 10 and 14 weeks (data not shown). The GMT of BMNA in the 10-week breast milk samples was similar in the withholding and immediate breastfeeding arms; 6.6 (95% CI: 5.2; 8.3) and 5.4 (95% CI: 4.3; 6.9), respectively.

The BMNA titer at 10 weeks was highly positively correlated with the serum MDNA titer at 6 weeks (p<0.001). When examining infants in the immediate feeding arm with low MDNA (< median titer of 109.4 in serum at 6 weeks of age), those with low BMNA (< median of 4 in breast milk collected 10 weeks post-partum) had higher seroconversion after two RV1 doses (44.4%) compared with those with higher BMNA (20.0%) (p-value 0.019) (Table 5), suggesting some inhibition by breast milk. At age 18 weeks after three RV1 doses, however, seroconversion was similar (38.9% vs. 38.0%) (p-value 1.0) (Table 5).

**Table 5:** Serum Anti-Rotavirus IgA Seroconversion in Immediate Breastfeeding Group, by MDNA and 10-Week BMNA titer level.

		Anti-Rotavirus IgA Seroconversion	Anti-Rotavirus IgA Seroconversion	Anti-Rotavirus IgA Seroconversion	Anti-Rotavirus IgA Seroconversion	Anti-Rotavirus IgA Seroconversion	Anti-Rotavirus IgA Seroconversion			
		Low BMNA Titer in Breast Milk at 10 weeks	Low BMNA Titer in Breast Milk at 10 weeks	Low BMNA Titer in Breast Milk at 10 weeks	High BMNA Titer in Breast Milk at 10 weeks	High BMNA Titer in Breast Milk at 10 weeks	High BMNA Titer in Breast Milk at 10 weeks	Difference in sero-conversion		
Timepoint	MDNA	n/N	%	95% CI	n/N	%	95% CI		95% CI	P-value
14 weeks (post 2 doses)	**Low**	16/36	44.4	28.2, 60.7	10/50	20.0	8.9, 31.1	24.4	4.8,44.1	0.019
14 weeks (post 2 doses)	**High**	9/31	29.0	13.1, 45.0	15/55	27.3	15.5, 39.0	1.8	-18.1,21.6	1.0
18 weeks (post 3 doses)	**Low**	14/36	38.9	23.0, 54.8	19/50	38.0	24.5, 51.5	0.9	-20.0,21.7	1.0
18 weeks (post 3 doses)	**High**	14/31	45.2	27.6, 62.7	18/55	32.7	20.3, 45.1	12.4	-9.0,33.9	0.353

MDNA Maternally Derived Neutralizing Antibodies; BMNA Breast Milk Neutralizing Activity

Low MDNA: below the median of 109.4 U/mL

Low BMNA: below the median of 4

### Safety assessment

Four SAEs were observed in each of the two study arms including two deaths in the withholding arm and one death in the immediate breastfeeding arm. An independent safety monitor found none of the SAEs related to the study vaccine.

## Discussion

In this study, withholding breastfeeding for one hour before and after the time of RV1 vaccine administration did not lead to increased anti-rotavirus IgA seroconversion compared with that seen with a breastfeed at the time of vaccination. In fact, to our surprise, IgA seroconversion after two RV1 doses at 6 and 10 weeks (the WHO recommended vaccination schedule) was significantly greater in immediately breastfed infants compared with those in whom breast milk was withheld, and this trend persisted after three RV1 doses although it did not remain statistically significant. Although serum rotavirus IgA is just one component of the immunological response to the vaccine and not necessarily a mechanistic correlate of protection, our findings suggest that substantially improved clinical protection is not likely with the strategy of withholding breastfeeding around the administration of RV1.

While immediate breastfeeding did not have a negative impact on IgA immune responses in our overall study population receiving two or three vaccine doses at young ages, some nuances need to be carefully considered. As has been demonstrated *in vitro* [5], our results from infants in the immediate feeding arm with low MDNA suggest that ingesting breast milk with higher BMNA at the same time as vaccine may reduce the subsequent immune response in some infants. Additionally, the GMT in seropositive infants at 14 weeks was higher in the withholding arm compared with the immediate arm. These latter two observations indicate that breast milk interference may occur in a subset of young infants, such as those with low MDNA and ingesting milk with high BMNA at the time of a particular dose.

It is unclear why IgA seroconversion in infants immediately breastfed tended to be higher than in those who were withheld from a feeding, opposite to our hypothesis. Buffering of gastric acid at the time of vaccine administration has been recognized as an important requirement for oral rotavirus vaccine ‘take’ [12]; RV1 is buffered with calcium carbonate. Our findings may suggest that additional buffering (as with a feed) may be worthwhile to investigate for some populations. This could be examined by evaluating a similar population but among whom formula feeding is more common, so as to isolate the effect of the possible additional buffering action of a concomitant feed. It is also possible, however, that this is a chance finding, possibly from differences in wild-type rotavirus exposure between the arms despite randomization. In a group of infants enrolled concomitantly as subjects in this study from a nearby area who did not receive rotavirus vaccine, background anti-rotavirus IgA seroconversion by age 18 weeks was 13.3% [8].

Our conclusion on lack of benefit of withholding a breastfeed is similar to that from a recent randomized trial from South Africa, which found that infants who were withheld a breastfeed for one hour before and after RV1 administration did not have improved immunogenicity (defined as ≥4-fold rise in serum anti-rotavirus IgA) after one or two doses of RV1 at 6 and 15 weeks, respectively, compared with infants in which unrestricted breastfeeding was encouraged; unlike our study, a documented immediate feed was not required and initially seropositive infants were included in the analysis [13]. Lack of overall benefit of withholding could be partially explained if a study population had very low levels of BMNA. Compared to BMNA from other populations reported from the same laboratory conducting our testing (but with a smaller number of samples for each population, and variable maternal and infant ages), the median titer of BMNA against RV1 in the 10-week samples from Pakistani mothers in our evaluation (titer 4) appears to be intermediate between that of US or Vietnamese (titer 2), Korean (titer 8) or South African mothers (titer between 8–16), and lower than Indian mothers (titer between 32–64); the infants in these studies had median age of about 3 months (range 4–29 weeks)[6, 7].

In conclusion, withholding breast feeding around the time of rotavirus vaccination does not appear to improve rotavirus vaccine immunogenicity in our study population. Alternative avenues to further augment immunogenicity and ultimately effectiveness of these vaccines in developing settings should be pursued.

## Supporting Information

S1 CONSORT Checklist (DOCX)

S1 Fig Serum anti-rotavirus IgA status in infants at age 18 weeks following a third dose of RV1, by their status at age 14 weeks following 2 doses of RV1. (TIF)

S1 Protocol (DOCX)

S1 Related Publication (PDF)
