# Correction: Impact of Withholding Breastfeeding at the Time of Vaccination on the Immunogenicity of Oral Rotavirus Vaccine—A Randomized Trial

**DOI:** 10.1371/journal.pone.0145568

**Published:** 2015-12-16

**Authors:** Asad Ali, Abdul Momin Kazi, Margaret M. Cortese, Jessica A. Fleming, SungSil Moon, Umesh D. Parashar, Baoming Jiang, Monica M. McNeal, Duncan Steele, Zulfiqar Bhutta, Anita K. M. Zaidi

There are errors in S1 Fig. Please view the correct S1 Fig below.

## Supporting Information

S1 Fig Serum anti-rotavirus IgA status in infants at age 18 weeks following a third dose of RV1, by their status at age 14 weeks following 2 doses of RV1. (TIF)
