# Supplementary figures and images for: Impact of Withholding Breastfeeding at the Time of Vaccination on the Immunogenicity of Oral Rotavirus Vaccine—A Randomized Trial
Source: PLoS One. 2015 Jun 2;10(6):e0127622. doi: 10.1371/journal.pone.0127622 (PMC4452702; doi:10.1371/journal.pone.0127622)

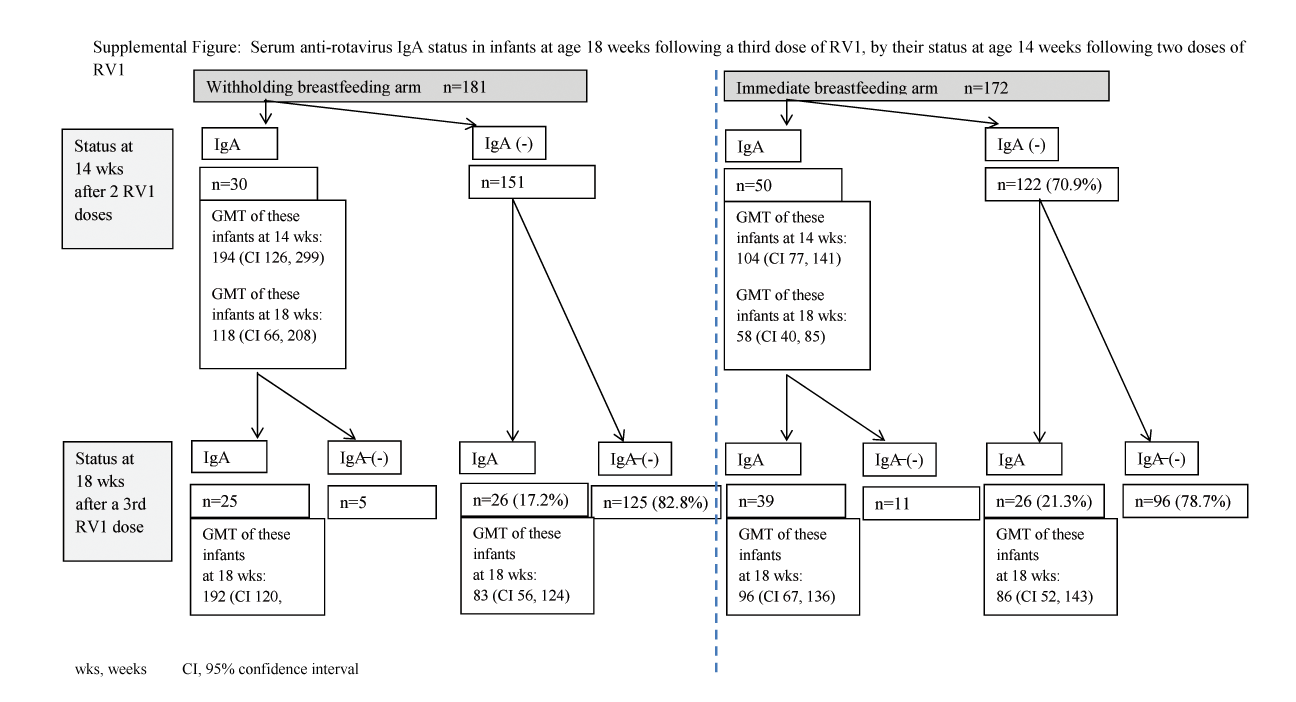

Supplement: S1 Fig — (TIF) [file pone.0127622.s002.tif]
