# Supplementary material for: Impact of Withholding Breastfeeding at the Time of Vaccination on the Immunogenicity of Oral Rotavirus Vaccine—A Randomized Trial
Source: PLoS One. 2015 Jun 2;10(6):e0127622. doi: 10.1371/journal.pone.0127622 (PMC4452702; doi:10.1371/journal.pone.0127622)
Supplement: S1 Protocol — (DOCX) [file pone.0127622.s003.docx]

**The Immunogenicity of Rotavirus Vaccine under Different Age Schedules and the Impact of Withholding Breast Feeding Around the Time of Vaccination on the Immunogenicity of Rotarix Vaccine.**

**Study Team:**

**Principal Investigator: S. Asad Ali MBBS, MPH^1^**

**Co-Investigator: A. Momin Kazi MBBS, MPH^1^**

**Senior Investigators: Anita Zaidi MBBS, SM^1^**

**Zulfiqar Bhutta MBBS, PhD^1^**

**Study Collaborators: Margaret Cortese, MD^2^**

**Umesh Parashar MBBS, MPH^2^**

**Duncan Steele, PhD^3^**

**Monica McNeal, PhD^4^**

**Baoming Jiang, PhD^2^**

**Jessica Fleming, PhD, MSc^3^**

**^1^Aga Khan University, Karachi, Pakistan**

**^2^Centers for Disease Control and Prevention, Atlanta, Georgia, USA**

**^3^Program for Appropriate Technology in Health (PATH)**

**^4^Cincinnati Children’s Hospital Medical Center**

**Sponsor: PATH**

2201 Westlake Ave, Suite 200, Seattle, WA 98121

**Summary**

Rotavirus is one of the most common causes of severe diarrhea, responsible for 40% of all diarrhea related deaths in children worldwide. Two vaccines against Rotavirus, Rotarix® and Rotateq® were licensed in many high and middle income countries in 2006, but lack of efficacy data in low income countries had prevented WHO from making a universal recommendation of their use until recently. Most high income countries follow an 8, 16 and 24 week (2, 4 and 6 months) schedule for administering routine vaccines (DPT, Hib etc.), while low income countries follow the 6, 10 and 14 week schedule for the same. The Strategic Advisory Group of Experts of WHO (SAGE) has recently recommended a two dose regimen of Rotarix® vaccination at 6 and 10 weeks of age in low income countries. The immunogenicity and efficacy of two dose regimen of Rotarix® at 8 weeks and 16 weeks (2 and 4 months of life) in high and middle income countries has been well documented; however, available data suggests that the 2 dose regimen administered at 6 and 10 weeks may be significantly less immunogenic and efficacious, perhaps due to lingering maternal rotavirus neutralizing antibodies in infants serum or due to higher level of rotavirus neutralizing antibodies in breast milk. Nonetheless, SAGE preferred to make the recommendation for Rotarix® immunization at 6 and 10 weeks of age (with 1^st^ and 2^nd^ DPT dose), as Rotarix® immunization at 10 and 14 weeks (with the 2^nd^ and 3^rd^ DPT dose) is considered programmatically difficult to implement at large scale, and a 3 dose regimen for Rotarix® immunization at 6, 10 and 14 weeks has significant cost implications.

One important question regarding the immunogenicity of oral rotavirus vaccines is that of concurrent breastfeeding at the time of vaccine administration. Recent evidence suggests that breast milk may have neutralizing activity against rotavirus, which may neutralize the rotavirus vaccine, thereby decreasing its immunogenicity. It is therefore important to determine if the immunogenicity of Rotarix® vaccine can be increased by temporarily withholding breastfeeding around the time of Rotarix vaccine administration.

We propose to conduct a study with two primary objectives: (A) to determine the immunogenicity of Rotarix® vaccine when administered at 6 and 10 weeks of life, at 10 and 14 weeks of life, and at 6, 10 and 14 weeks of life. (B) To determine the role of withholding breastfeeding for one hour before and after Rotarix® vaccine administration on the immunogenicity of Rotarix® vaccine administered in 6, 10 and 14 week regimen.

1. **INTRODUCTION**
   1. **Background**

Rotavirus is the most common cause of severe gastroenteritis in children<5 years of age, accounting for an estimated 2.4 million hospital admissions and611,000 deaths each year(1-4). Because of this tremendous global burden of rotavirus, vaccine development and introduction has been a high priority for several international agencies, including the World Health Organization (WHO) and the Global Alliance for Vaccines and Immunization (GAVI)(5). In 2006, two rotavirus vaccines—Rotateq® (Merck and Co., Inc.) and Rotarix® (GlaxoSmithKline) were licensed for use in many countries. Pre-licensure clinical trials of each of these vaccines have demonstrated high efficacy (85-98%) against severe rotavirus disease and a good safety profile(6;7). These pivotal clinical trials were conducted in middle and high-income countries and need for similar studies in low income countries of Asia and Africa, where 85% of the rotavirus associated mortality occurs, was highlighted by WHO and GAVI. In response, multiple efficacy trials are being conducted in low income countries, and the expected time line of their results is summarized in table 1.

|  |  | 2008 | 2009 | 2010 |
| --- | --- | --- | --- | --- |
| GSK  Vaccine,  Rotarix®  (Human,  Monovalent | Asia |  |  | Bangladesh  effectiveness  study |
|  | Africa | South Africa  Interim  Analysis | Malawi &  South Africa  Final Analysis | Malawi &  South Africa  Extended  Follow-up  Analysis |
| Merck  Vaccine,  Rotateq®  (Bovine,  Reassortant,  Multivalent) | Asia |  | Bangladesh &  Vietnam  Final Analysis |  |
|  | Africa |  | Ghana, Kenya,  & Mali  Final Analysis |  |

*Steele et al. Data presented in 8th International Rotavirus Symposium, Turkey, 2008

The initial results of a double-blind, randomized, multicenter, placebo-controlled phase III efficacy trial of Rotateq® conducted in Africa and Asia have recently become available. In this study, 3 doses of vaccine or placebo were given at 6, 10 and 14 weeks to infants in participating sites (8).  The study was designed to separately analyze the combined results for the sites in 3 countries in Africa (Ghana, Kenya and Malawi) and the combined results for the sites in 2 countries in Asia (Bangladesh and Vietnam) (9).  The efficacy of a 3-dose regimen of the vaccine against severe rotavirus gastroenteritis during the first year of follow-up was 64.2% in Africa (95% CI: 40.2.0-79.4%) and 51.0% in Asia (95% CI, 12.8 – 73.3%).

The results from a phase III trial of Rotarix® in South Africa and Malawi comparing the efficacy of two dose regimen of Rotarix® administered at 10 weeks and 14 weeks of life with the three dose regimen at 6 weeks, 10 weeks, and 14 weeks have also recently been reported (10). Overall efficacy of the 2-dose schedule was 58.7% (95% CI, 35.7–74.0%), with 72.2% (95% CI, 40.4–88.3%) efficacy in South Africa and 49.2% (95% CI, 11.1–71.7%) in Malawi; overall efficacy of the 3-dose schedule was 63.7% (95% CI, 42.4–77.8%), with 81.5% (95% CI, 55.1–93.7%) in South Africa and 49.7% (95% CI, 11.3–72.2%) in Malawi (9). On June 5, 2009, based on the available data, the Strategic Advisory Group of Experts of WHO (SAGE) made a policy recommendation of inclusion of rotavirus vaccines in all low-income countries, especially where the proportion of mortality due to diarrhea is > 10%(10).

After the recommendation of use, the next step is to decide the optimum age and dosage schedule for administering this vaccine in low-income countries. In countries using a 8, 16 and 24 week (2-month, 4-month, 6-month) schedule for delivering DTP vaccine (for example, in Latin America), the effectiveness of 2 doses of Rotarix given at 2 months and 4 months has been demonstrated(6).The efficacy data presented to SAGE for Rotarix in populations where there is high mortality among children incorporated a 3-dose schedule at 6 weeks,10 weeks and 14 weeks of age, and a 2-dose schedule at 10 weeks and 14 weeks. There is no evidence for the efficacy or effectiveness of Rotarix vaccine with a 2-doseschedule at 6 weeks and 10 weeks of age. A study in South Africa by Steele *et al* suggests that 6 and 10 week schedule maybe less immunogenic than 10 and 14 week schedule, presumably because of interference by maternal antibody(11). Despite concerns that a 2-dose schedule administered on time at 6 weeks and 10 weeks may be less effective, SAGE recommends that 2 doses of Rotarix should be administered with the first and second doses of DTP. The aim of this recommendation was to ensure maximum immunization coverage with the Rotarix vaccine, since the two dose regimen of 10 weeks and 14 weeks was not considered practical or programmatically feasible. For example, with a recommendation of Rotarix immunization at the time of second and third DPT dose (at 10 weeks and 14 weeks of age), many infants presenting at 8 weeks for the initial vaccine visit will be refused Rotarix immunization. However, we believe that the current SAGE recommendation of immunizing with two dose regimens at 6-weeks and 10-weeks needs to be underpinned by solid evidence demonstrating immunogenicity of this regimen compared to 10 and 14 weeks regimen and 6 weeks, 10 weeks, and 14 weeks regimen. This data is critical for ensuring maximum public health benefit from rotavirus vaccination program.

SAGE also does not make any recommendation about withholding or not withholding breast feeding at the time of Rotarix® administration due to lack of clear evidence. Recent studies have shown that breast feeding practices may be an important factor contributing towards the lower immunogenicity and efficacy of Rotarix® in low income countries. A review article by Patel et al summarizes the current literature on this subject very nicely(12). Breast milk contains IgA antibodies that can neutralize rotavirus, as well as receptor analogues that can absorb to the virus and inhibit its attachment(13). These factors are greatest in the first days after parturition and decrease as breastfeeding progresses. In an *in vitro* study where breast milk was mixed with an equal volume of live virus, about 30% of the breast milk samples were able to neutralize 99% of virus and 60% of the breast milk samples could neutralize 90% of virus(14). These data suggest that if an infant had breast milk in its mouth or stomach at the time the vaccine was administered, this could diminish vaccine response.

The effect of breastfeeding on vaccination has been explored in the large-scale field trials of the GSK and Merck vaccines in populations in high and middle income countries. No differences in vaccine efficacy were noted between mothers who self-reported breastfeeding compared with those who did not(6;7). However, none of these studies examined the critical parameter of specific time interval between breast feeding and the administration of vaccine. If the vaccine was administered at a time when the infant had not recently been feeding, the vaccine strain could pass unimpeded to the gut and begin replicating. However, if the infant was being breast fed at the very time of vaccination, its mouth and gastrointestinal tract could become *in vivo* sites for virus neutralization and the vaccine might be expected to have a diminished effect. No studies have been conducted to date to determine whether the time interval between breastfeeding and immunization has any effect on the immune response to the vaccine(15). In order to maximize the public health impact of rotavirus vaccine, it is important to study the role of withholding breast feeding around the time of Rotarix® administration on the resultant immunogenicity.

We are proposing to conduct a clinical trial to answer the two critical questions related to rotavirus immunization program in low-income countries- (i) optimum age and dosage schedule of administrating rotavirus vaccine in countries following 6, 10 and 14 week schedule for administrating DTP and (ii) the impact of withholding breast feeding at the time of rotavirus vaccination at the time of rotavirus vaccine. This clinical trial will be conducted at an urban community in Pakistan. Conclusions of this trial will help the WHO decision makers in making evidence based future recommendations of rotavirus vaccine use in low income countries of the world.

1. **PROJECT OBJECTIVES**
   1. **Primary Aim 1**

- To compare the immunogenicity of live oral rotavirus vaccine (Rotarix®) when administered at 6 and 10 weeks of life, and 6, 10 and 14 weeks of life, using serum rotavirus IgA seroconversion as the marker of vaccine induced immunogenicity.
  - 1. **Secondary Aim**
- To compare the immunogenicity of live oral rotavirus vaccine (Rotarix®) when administered at 6 and 10 weeks of life and 10 and 14 weeks of life, using serum rotavirus IgA seroconversion as the marker of vaccine induced immunogenicity.

- To determine the effect of maternally derived serum rotavirus neutralizing antibodies on the immunogenicity of rotavirus vaccine by determining if infants with high maternally-derived serum rotavirus neutralizing antibodies have lower rates of IgA seroconversion after vaccine compared to infants with lower maternally-derived serum rotavirus neutralizing antibodies.
  1. **Primary Aim II**
- To compare the immunogenicity of Rotarix vaccine (6,10,and 14 weeks regimen) in infants breast fed at the time of vaccine administration, with infants whose breast feeding is withheld for one hour before and after vaccine administration.
  - - 1. **Secondary Aim II**
- To determine the effect of rotavirus neutralizing activity in breast milk on the immunogenicity of Rotarix® vaccine in infants by determining if infants exposed to breast milk with high levels of rotavirus neutralizing activity have lower rates of IgA seroconversion after vaccine, compared to infants exposed to breast milk with lower levels of rotavirus neutralizing activity.
- To determine the correlation of oral rotavirus IgA with serum rotavirus IgA in infants.
  1. **Primary Aim 3:**
- To determine baseline seroconversion rate for rotavirus in children in Pakistan by 18 weeks of age

**STUDY COMPONENTS**

The study is divided in three components

1. **Study A: Comparison of the immunogenicity of Rotarix® vaccine when administered at 6 and 10 weeks of age, 10 and 14 weeks of age, and 6, 10 and 14 weeks of age.**
2. **Study B: Impact of withholding breastfeeding around the time of Rotarix® vaccination on the immunogenicity of Rotarix® vaccine.**
3. **Study C: To evaluate baseline seroconversion rate for rotavirus among children in the first 18 weeks of life.**

The specific design elements of each of these three studies are described separately.

1. **research design and methods**

**Study A: Comparison of the immunogenicity of Rotarix® vaccine when administered at 6 and 10 weeks of age, 10 and 14 weeks of age, and 6, 10 and 14 weeks of age.**

- 1. **Study Site**

The study will be conducted at one site in an urban- squatter settlement area, Ali Akber Shah Goth (AG) in Karachi where the Aga Khan University’s Department of Paediatrics and Child Health has demographic surveillance in place, including pregnancy and newborn surveillance. The annual birth cohort in the area is approximately 1600 infants. The site is about 30 minutes driving distance from the Aga Khan University Medical College and Hospital campus. A GIS map of the site is available. The Department will provide EPI immunizations at this location.


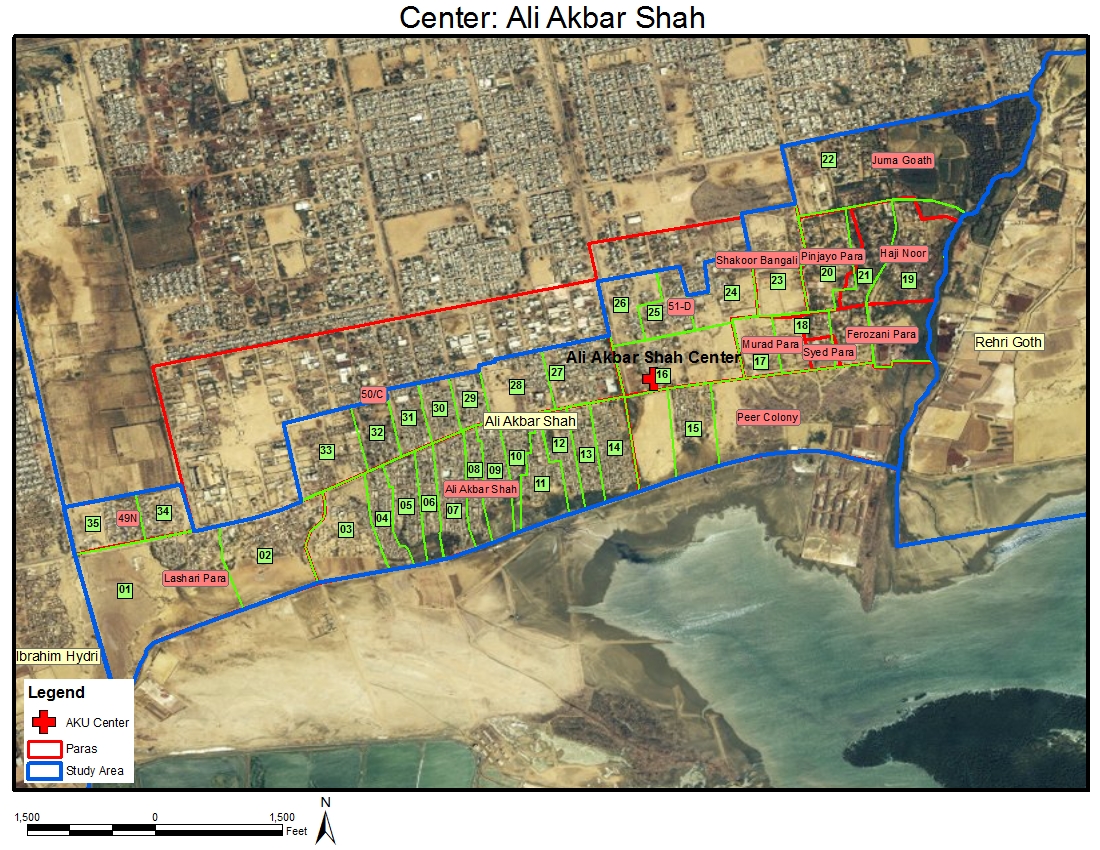


- 1. **Study Design**

This study will compare the immunogenicity of Rotarix® vaccine when administered in three different schedules:

**Arm 1:** will receive the routine EPI vaccines according to the usual schedule in Pakistan (6, 10 and 14 weeks of age) and the rotavirus vaccine at 6 and 10 weeks of age

**Arm 2:** will receive the routine EPI vaccines according to the usual schedule in Pakistan (6, 10 and 14 weeks of age) and the rotavirus vaccine at 10, and 14 weeks of age

**Arm 3:** will receive the routine EPI vaccines according to the usual schedule in Pakistan (6, 10 and 14 weeks of age) and the rotavirus vaccine at 6, 10, and 14 weeks of age

- 1. **Sample Size Calculations**

Target enrollment would be 600 infants; 200 in each of the arms 1,2 and 3. Assumptions used for calculation of sample size are estimated seroconversion rate in arm 1 of35% (11), and in arm 2 and 3 of 52.5%(investigational), power at 0.85, and alpha error of 0.05, using a two-tailed t-test & Chi-square test and assuming 20% drop out and 5 % expected IgA seropositive at 6 weeks. The estimated sample size needed in each group is 191.

**3.4 Inclusion Criteria**

- - 6 weeks 0 days to7 weeks 6 days age at the time of enrollment.
  - Healthy infant free of chronic or serious medical condition as determined by history and physical exam at time of enrollment into in the study.
  - Written informed consent obtained from the parents or guardians.
  1. **Exclusion Criteria**
- Hypersensitivity to any of the vaccine components.
- Use of any investigational drug or vaccine other than the study vaccine within 30 days of first dose of study vaccine or during the study.
- Use of any immunosuppressive drugs.
- Previous intussusceptions or abdominal surgery.
- Enrolment in any other trial (Simplified Antibiotic Therapy for Neonatal Sepsis Trial, Management of Omphalitis Trial).
- Birth weight less than 1500 grams; or if birth weight is unknown, weight less than 2000 grams on or before 28 days.
- Immunoglobulin and/or blood products use since birth or during the study period.

Rotavirus vaccine administration should be postponed in subjects who, per the clinician’s assessment:

- - Have vomited during each of the last three feedings
  - Are recommended for hospitalization at the visit
  - Are suffering from acute febrile illness. Acute febrile illness is defined as temperature ≥ 38.0^o^ measured by rectal or axillary thermometer (Meet WHO criteria for moderate to severe dehydration (= those needing ORS rehydration in the facility or intravenous rehydration).
  - EPI vaccinations will be provided or postponed as per the clinician’s assessment and according to standard practice.
  1. **Elimination Criteria During the Study**
- Rotavirus IgA seropositivity in the blood specimen obtained at age 6 weeks, if the results become available before the time additional rotavirus vaccine doses are due to be given or additional blood draws or other samples are due to be obtained. These children will not be included in the final analysis, since they are already seropositive and cannot seroconvert due to rotavirus vaccine. If the 6-week IgA results become available before the time additional rotavirus vaccine doses are due or study samples are scheduled to be obtained, these children will not receive further rotavirus vaccine doses or undergo further blood draws or study sample collection; however, we will ensure that they continue to receive the routine EPI vaccines.
- Infants who develop intussusceptions during the study will not receive additional doses of rotavirus vaccine.
- Infants who have a hypersensitivity reaction due to rotavirus vaccine and who have not yet received their final dose of rotavirus vaccine.
  1. **Contraindication of the Rotavirus Vaccine**
     - - Hypersensitivity reaction due to the rotavirus vaccine
       - Infants who develop intussusceptions during the study
  2. **Recruitment Process**
  - Eligible infants will be recruited through the community health workers on their routine surveillance in the Ali Akber Shah Goth.
  - The CHWs will approach the families who have an infant between 4 to 6 weeks of age. Parents or caretaker will be briefed about the study and visits to be made at PHC for vaccination and blood draws.
  - If the parent/caretaker is interested in the study, CHW will evaluate the infant by enrollment check list.
  - Parents or caretaker of eligible infants will be provided transportation for visits to be made at PHC for enrollment, vaccination and blood draws.
  - At 6 weeks of age, infant of interested parents will be brought to the PHC where infants will undergo history and physical examination. Consent will be obtained and Infants will be enrolled if they meet the eligibility criteria.
  - Eligible Infants will be randomly allocated to one of the three groups using randomly generated computer assignments, allocated in sealed envelopes to be opened at the time of enrolment.
  - Baseline information on socio – demographics background, pregnancy, prenatal and delivery details, medical and vaccination history will be recorded.

**Monitoring Serious Adverse Events**

**Serious Adverse Event:** A serious adverse event (SAE) is defined as an adverse event that meets one of the following:

- Death
- Requires inpatient hospitalization or prolongation of existing hospitalization
- Life threatening (subject at immediate risk of death).
- Results in a persistent or significant disability or incapacity
- Important medical events that may not results in death, be life threatening, or require hospitalization may be considered SAEs when, based upon appropriate medical judgment, may jeopardize the subject and may require medical or surgical intervention to prevent one of the outcomes listed in this definition.

All serious adverse events occurring at anytime during the study will be captured.

**Relationship to Study Vaccines**

The clinician’s assessment of the relationship of a SAE to the study vaccine (Rotarix) is part of the documentation process, but it is not a factor in determining what is or is not recorded in the study. If there is any doubt as to whether a clinical observation is an SAE, the event will be recorded. All SAEs must have their relationship to Rotarix assessed using the terms “related” or “not related”. The following guidelines will be used:

Related – the event is temporally related to the administration of the vaccine and no other etiology explains the event

Not related – the event is temporally independent of the vaccine and/or the event appears to be explained by another etiology.

**Reporting Procedures**

All SAES occurring during the study period must be reported to the study sponsor, even if the investigator considers that the SAE is not related to treatment. The study clinical staff will complete a SAE form within the following timelines of such events.

- All deaths and immediately life–threatening events, whether related or unrelated to study treatment will be recorded on the SAE form and sent to PATH via email ([jfleming@path.org](mailto:jfleming@path.org)) by the site within 24 hours of becoming aware of the event
- SAEs other than death and immediately life-threatening events, regardless of relationship, will be reported to PATH via email ([jfleming@path.org](mailto:jfleming@path.org)) by the site within 72 hours of becoming aware of the event.

**Halting Rules**

Given the worldwide experience with Rotarix, a licensed vaccine in Pakistan, no safety issues are anticipated which would result in the suspension of enrollment in the study. However, as this study will be conducted in an impoverished population in the developing world, should a serious adverse event occur which is likely related to vaccination with Rotarix, the decision whether the study should continue per protocol, proceed with caution, be further investigated, be discontinued, or be modified and then proceed will be made by the Aga Khan IRB, in consultation with the PI, PATH and CDC. However, no rules will be pre-specified to define these conditions. If the study is halted, parents/guardians of subjects will be contacted immediately explaining why the study was halted and the implications for the safety and/or protection of their child against rotavirus.

**Safety Oversight by Independent Safety Monitor**

The Independent Safety Monitor **(**ISM) will be a physician with relevant expertise whose primary responsibility is to provide independent safety monitoring in a timely fashion. This is accomplished by review of the SAEs, with follow-up through resolution. The ISM will evaluate individual and cumulative participant data when making recommendations regarding the safe continuation of the study. The ISM will not be under direct supervision of the investigator and will not be involved in the study in any other capacity.

The ISM will act as the sole independent monitor. As such, PATH will confirm prior to any review of data and at least annually that no conflict of interest exists. Interests that may create a potential conflict of interest should be disclosed to PATH prior to review of any data. PATH will determine if the relationship is in conflict or gives the appearance of a conflict such that the individual should not serve as the ISM. PATH will determine how to handle such potential conflict. PATH may dismiss an ISM in the event of unmanageable potential conflict.

**Study Materials for ISM Review**

The primary focus of the ISM is to independently review all serious adverse events. As the sole monitor, the ISM will evaluate all such events against the known safety profile of the rotavirus vaccine. Clinical and laboratory data, clinical records, and other study-related records should be made available for ISM review. If necessary, special reports will be prepared by the investigator.

**Reports from the ISM**

The following reports may be submitted by the ISM: review report and immediate action report.

**Review Report**

The ISM will communicate in writing his/her findings and any concerns and recommendations to the Sponsor representative at PATH. If the ISM does not concur with the assessment provided by the local investigative staff, the written report will then be forward to the local PI in Pakistan, who must file a copy in the study files and submit the report as per local IRB policy.

**Immediate Action Report**

The ISM will notify the Sponsor representative of any findings of a serious and immediate nature including any recommendations to discontinue all or part of the trial, and the Sponsor representative will immediately inform the PI, other PATH investigators, and CDC. In additional to any verbal communications, recommendations to discontinue or substantially modify the design or conduct of a study must be conveyed in writing on the day of the ISM review.

- 1. **Study schema**

CHWs will recruit healthy infants between 4- 6 weeks of age on their routine surveillance

CHW will do the assessment of the infant through enrollment check list

Parents or caretaker of eligible infants will be provided transportation for required visits

Primary Health Care Center

Medical Officer

History and PE to assess eligibility

**Eligible Not eligible**

Consent requested by study

Physician

Exit

**Consent declined**

**Consent given**

Randomization into Arm 1, 2, 3

- 1. **Vaccination and Blood Draw Schedule.** Blood samples will be obtained at the scheduled visits just prior to the administration of vaccines.

**Arm 3**

Rotarix® Vaccination

n= 200

**Arm 2**

Rotarix® Vaccination

n= 200

**Arm 1**

Rotarix® Vaccination

n= 200

**Visit at 6 weeks**

- Pre vaccine blood draw
- EPI vaccination
- 1^st^ dose Rota virus vaccination

**Visit at 6 weeks**

- Pre vaccine blood draw
- EPI vaccination
- 1^st^ dose Rota virus vaccination

**Visit at 6 weeks**

- Pre vaccine blood draw
- EPI vaccination

**Visit at 10 weeks**

- EPI vaccination
- 1^st^ dose Rota virus vaccination

**Visit at 10 weeks**

- EPI vaccination
- 2^nd^ dose Rota virus vaccination

**Visit at 10 weeks**

- EPI vaccination
- 2^nd^ dose Rota virus vaccination

**Visit at 14 weeks**

- EPI vaccination
- 3^rd^ dose Rota virus

**Visit at 14 weeks**

- EPI vaccination
- 2^nd^ dose Rota virus vaccination
- sample on day’s 3 and 7 after vaccination

**Visit at 14 weeks**

- Blood draw
- EPI vaccination

**Visit at 18 weeks**

- Blood draw

**Visit at 18 weeks**

- Blood draw

**Visit at 18 weeks**

- Blood draw
  1. **Enrollment Time Period**

Enrolment will be completed over a period of one year so that all the seasons are covered. The rotavirus infections in Karachi are seasonal, with peaks in both summer and winter months which can influence baseline seroconversion. Refer to section 4.5

- 1. **Enrollment Schedule**

| ***Treatment Group*** | ***Number of Subjects*** | **Youngest (Y): 6wk, 0dy**  **Oldest(O): 7wk, 6dy** | **Y: 10wk, 0dy**  **O: 12wk, 6dy** | **Y: 14wk, 0dy**  **O: 17wk 6dy** |
| --- | --- | --- | --- | --- |
| Arm 1 | 200 | Standard EPI + Rotarix® | Standard EPI + Rotarix® | Standard EPI |
| Arm 2 | 200 | Standard EPI | Standard EPI + Rotarix® | Standard EPI + Rotarix® |
| Arm 3 | 200 | Standard EPI + Rotarix® | Standard EPI + Rotarix® | Standard EPI + Rotarix® |

*Standard EPI in Pakistan is OPV plus BCG at birth; DPT-Hep-B-Hib and OPV at 6, 10, and 14 weeks, and measles at 9 months and second year of life

- 1. **Sampling Collection and Handling**

3 ml of blood sample will be obtained by a trained health worker according to WHO standard procedures. Blood draws will be done at 6, 14 and 18 weeks of age. Blood samples from this study will be used only for rotavirus IgA and neutralizing antibody assay testing. Samples will be stored with a unique identification number specific for the study child.

- 1. **Lab Assay**

| ***6wks*** | ***10wks*** | ***14wks*** | ***18wks*** |
| --- | --- | --- | --- |
| **All arms:**  Rotavirus IgA (Serum)  Rotavirus Neutralizing Antibody (Serum) |  | **Arm 1 only**  Rotavirus IgA (Serum) | **All Arms**  Rotavirus IgA (Serum) |
|  |  |  |  |
|  |  |  |  |

- 1. **Laboratory Training for Rotavirus antibody assays**

We will make arrangements with the laboratory of Dr. Monica McNeal’s lab in Cincinnati, OH, USA to train one senior laboratory technician from our laboratory in performing rotavirus IgA. Since rotavirus neutralizing antibody assays are complicated and the number of samples that need to be tested for neutralizing assay is small, samples will be shipped to Dr. Monica McNeal’s lab in Cincinnati, OH for testing of neutralizing antibodies.

- 1. **Data Entry, Cleaning and Management**

All completed case report forms (CRFs) will be edited by the field RA at the PHCs and a second quality check on all forms will be done by the data RAs at the Data Management Unit (DMU). A data entry program (database and entry screens) will be created in MS Access 2007. Program will contain logical checking and will support double data entry. Dual entry error checking will be done on weekly basis and error report will be generated for corrections. After dual entry checking the data will undergo second phase of logical checking before analysis.

The database will reside on a central computer managed by the Data Manager. All data entry operators will access the program through the local network. The database will be regularly backed up every day on site and weekly off site on an external device and on the main university servers. All hard copies of the forms will be stored in two different locations: the first temporary location will be in the room where quality check, data entry and cleaning operations take place; permanent storage will be in a document storage room. Both storage rooms will be locked and moving of forms will be tracked using an Excel sheet.

- 1. **Data Analysis of Demographics**

The mean, range standard deviation of height in cm, weight in kg and of age in weeks will be calculated per group. The racial and gender composition per group will be presented.

- 1. **Analysis of immunogenicity**

Seropositivity will be defined as an anti-rotavirus IgA concentration ≥20 U/ml. Seroconversion will be defined as the detection of anti-rotavirus IgA concentration ≥ 20 U/ml in infants seronegative for anti-RV IgA at age 6 weeks before vaccination. The primary analyses will be performed on the according to protocol (ATP) cohort. Rotavirus IgA seroconversion rate (proportion of infants who have rotavirus IgA antibodies in serum sample) by age 18 weeks will be determined for infants in each arm who were rotavirus IgA –negative at 6 weeks of age. The primary endpoint will be the comparison of the rotavirus IgA seroconversion rate at age 18 weeks in arm 3, compared to the greatest IgA seroconversion rate for arm 1 (either the result from the 14 week samples or the 18 week samples). This latter aspect is included in order to provide the most relevant comparison between arms 1, 2 and 3 because both times since last dose of rotavirus vaccine and age at last blood draw can affect seroconversion estimates. Seroconversion rates will be compared between arm 1 and arm 3 using a Chi-square test. Confidence intervals (95%) for the differences in seroconversion rates will also be calculated. Rotavirus IgA GMC (geometric mean concentration) will be calculated and compared between the arms using a t-test. As described above for seroconversion, this would be at age 18 weeks for arms 1 and 3, and the highest result from the 14- and 18 week sample for arm 2.

- 1. **Role of Maternally derived Rotavirus Neutralizing antibodies in the immunogenicity of Rotavirus vaccine:**

The distribution of the level of maternally-derived rotavirus antibodies in the 6 week serum samples (among those infants who were rotavirus IgA negative at age 6 weeks and completed the study ATP) will be examined. The independent effect of the level of maternally-derived neutralizing antibody level on the rate of rotavirus IgA seroconversion by age 18 weeks will be assessed using logistic regression. Vaccine schedules will be included {(Arm 1 vs. Arms 3(primary) and Arm 1 vs. Arm2 (secondary)} as an independent variable and effect modification by neutralizing antibody level will be examined. Exploratory analyses will be performed for correlation between maternally-derived neutralizing antibody level and rotavirus IgA level at age 18 weeks among the two vaccine schedule categories

**Study B: Impact of withholding breastfeeding around the time of Rotarix® vaccination on the immunogenicity of Rotarix® vaccine.**

- 1. **
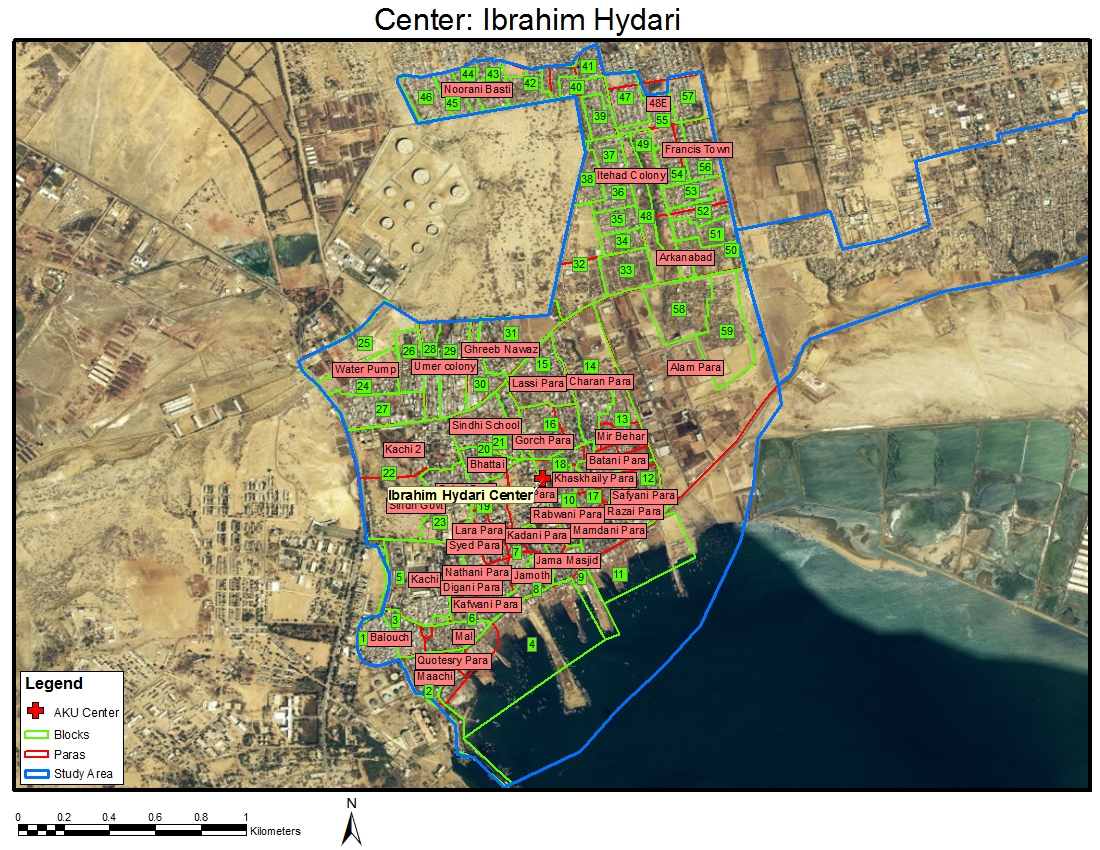
Study Site**

The study will be conducted at one site in an urban- squatter settlement area, Ibrahim Haidry in Karachi where the Aga Khan University’s Department of Paediatrics and Child Health has demographic surveillance in place, including pregnancy and newborn surveillance. The annual birth cohort in the area is approximately 1800 infants. The site is about 30 minutes driving distance from the Aga Khan University Medical College and Hospital campus. A GIS map of the site is available. The Department will provide EPI immunizations at this location.

- 1. **Study Design**

This study will compare the immunogenicity of Rotarix ® vaccine in relation to the timing of breast feeding. The two arms of the study will receive the EPI vaccines according to the usual schedule in Pakistan at ages 6, 10 and 14 weeks and Rotarix® vaccine at 6, 10, and 14 weeks of age. In arm 1, mothers would be asked to breast feed immediately before administration of the Rotarix dose. In arm 2, mothers would be asked to withhold breast feeding for one hour before and one hour after each Rotarix®dose.

Infants potentially eligible for the study will be identified by the CHWs during their routine household surveillance, and will be brought to the PHC at 4 weeks of age or at the time of first contact with the baby. In the PHC, infants will undergo history and physical examination by the study medical officer to determine eligibility. Study procedures will be explained to the mothers, including the need to withhold breastfeeding or to breastfeed at time of the vaccine doses, depending on the study arm. Consent will be asked from the parents of infants who are eligible. After obtaining consent, infants will be randomized into the breast feeding with holding or immediate breastfeeding arms.

Mothers who earlier provided consent and were randomized to the immediate breastfeeding group will be picked up at their home approximately 1 hour before the expected immunization time and instructed not to breastfeed until the indicated time. They will be taken to the clinic, infant blood sample will be obtained, routine EPI immunization will be given and mother will be asked to breastfeed the baby for at least 20 minutes. CHW staff will observe the breastfeeding. The Rotarix vaccine will be given, and then baby will be observed for 20 minutes to document if any vomiting occurred.

Mothers who earlier provided consent and were randomized to the withholding breastfeeding group will be picked up at their home approximately 1 hour before the expected immunization time and instructed not to breastfeed until the indicated time. They will be taken to the clinic, infant blood sample will be obtained, routine EPI immunization will be given, Rotarix vaccine will be given, and then mother will be asked to not breastfeed for one hour after the dose. CHW staff will observe the mother and infant during this period and document withholding of breastfeeding and to document if any vomiting occurred. After the 1 hour period, the mother will be able to breastfeed the infant if desired.

On the day of each vaccine visit, the mother will be asked to express 10 ml of breast milk into a study container while the CHW is present in the home to assist as needed or in the clinic and to refrigerate the sample as soon as it is expressed.

At age 6, 14 and 18 weeks, in breast feeding withholding arm 1 ml of oral fluid (OF) will be taken from infants in order to determine the correlation of IgA in OF and serum. Arm 1 provides an ideal setting to assess the value of non-invasive OF assays for rotavirus immune response, avoiding the potential contamination from breast milk.

- 1. **Sample Size consideration:**

Target enrollment would be 400 infants (200 each in each group) to be able to detect a difference in seroconversion rate of at least 17.5% between the withholding breastfeeding group and the immediate breastfeeding group, if such a difference truly exists.

Assumptions used for calculation of sample size are an estimated seroconversion rate in the immediate breastfeeding infants of 45% at age 18 weeks and in the withholding breastfeeding infants of 62.5% at age 18 weeks; power at 0.85, and a two-sided alpha of0.05. Using a Chi-square test and assuming 20% drop out and 5% baseline IgA seropositivity at 6 week, the estimated sample size needed in each group is 191.

- 1. **Study schema**

CHWs will identify healthy infants younger than 5 weeks of age on their routine surveillance using the enrollment checklist

Parents or caretaker of eligible infants will be provided transportation for required visits to PHC

Child referred to PHC for enrollment and randomization at 5 weeks

History and physical examination by PHC Medical Officer to assess eligibility

Consent asked from parents of eligible infants

**Eligible Not eligible**

Exit

Consent by study

Physician

**Consent declined**

**Consent given**

Randomized in withholding breast feed or immediate breast feeding arm

- 1. **Breastfeeding Immunogenicity Study**

**Withholding Breastfeeding Arm (Arm 2)**

n= 200

**Immediate Breastfeeding Arm (Arm 1)**

n=200

**Visit at 6 weeks**

- Breast milk sample obtained from mother
- Pre vaccine blood sample obtained from infant
- EPI vaccines given
- 1^st^ dose Rota virus vaccine
- Breast feed immediately before vaccination

**Visit at 6 weeks**

- Breast milk sample obtained from mother
- Pre vaccine blood and oral fluid samples obtained from infant
- EPI vaccines given
- 1^st^ dose Rota virus vaccine
- One hour withholding of breast feed before and after vaccination

**Visit at 10 weeks**

- Breast milk sample obtained from mother
- EPI vaccines given
- 2^st^ dose Rota virus vaccine
- One hour withholding of breast feed before and after vaccination

**Visit at 10 weeks**

- Breast milk sample obtained from mother
- EPI vaccines given
- 2^st^ dose Rota virus
- Breast feed immediately before vaccination

**Visit at 14 weeks**

- Breast milk sample obtained from mother
- 3^rd^ dose Rota virus vaccination
- Blood sample obtained from infant
- EPI vaccines given
- Breast feed immediately before vaccination

**Visit at 14 weeks**

- Breast milk sample obtained from mother
- 3^rd^ dose Rota virus vaccination
- Blood and oral fluid samples obtained from infant
- EPI vaccines given
- One hour withholding of breast feed before and after vaccination

**Visit at 18 weeks**

- Blood sample obtained from infant

**Visit at 18 weeks**

- Blood and oral fluid samples obtained from infant
  1. **Lab Assay**

| **Youngest (Y): 6wk, 0dy**  **Oldest(O): 7wk, 6dy** | **Y: 10wk, 0dy**  **O: 12wk, 6dy** | **Y: 14wk, 0dy**  **O: 17wk 6dy** | **Y: 18wk, 0dy**  **O: 22wk, 6dy** |
| --- | --- | --- | --- |
| ***Infant serum*** |  | ***Infant serum*** | ***Infant serum*** |
| Rotavirus IgA  Neutralizing Antibodies |  | Rotavirus IgA | Rotavirus IgA |
| ***Oral fluid(withholding group only )*** |  | ***Oral fluid(withholding group only )*** | ***Oral fluid(withholding group only )*** |
| Rotavirus IgA |  | Rotavirus IgA | Rotavirus IgA |
| ***Breast milk sample*** | ***Breast milk sample*** | ***Breast Milk sample*** |  |
| Rotavirus IgA  Rotavirus Neutralizing Activity | Rotavirus IgA  Rotavirus Neutralizing Activity | Rotavirus IgA  Rotavirus Neutralizing Activity |  |

- 1. **Sampling Collection and Handling**

Same as section 3.13

- 1. **Laboratory Training and testing for rotavirus antibody assays**

Serum rotavirus IgA and rotavirus neutralizing antibody testing will be done as described in section 3.15. Assessment of rotavirus antibody and rotavirus neutralizing activity in breast milk, and rotavirus antibody in oral fluid will be done at CDC- Dr. Baoming Jiang lab, who is the pioneer of this assay.

- 1. **Data Entry, Cleaning and Management**

Same as section 3.16

- 1. **Data Analysis of Demographics**

Same as section 3.16

- 1. **Analysis of immunogenicity**

Seropositivity will be defined as an anti-rotavirus IgA concentration ≥20 U/ml. Seroconversion will be defined as the detection of anti-rotavirus IgA concentration ≥ 20 U/ml in infants seronegative for anti-RV IgA at age 6 weeks before vaccination. The primary analyses will be performed on the according to protocol (ATP) cohort. The primary endpoint will be the comparison of the rotavirus IgA seroconversion rate at age 18 weeks between the immediate breastfeeding and the withholding breastfeeding groups, using a Chi-square test. Confidence intervals (95%) for the difference in seroconversion rates will also be calculated. Rotavirus IgA GMC (geometric mean concentration) at age 18 weeks will be calculated and compared between the arms using a t-test. Similar analyses will be performed comparing the seroconversion rate at age 14 weeks between the two arms.

The correlation between serum and OF IgA will be determined (1) by the concordance in detecting seroconversion, as defined above, at 14 and 18 weeks and (2) the correlation between antibody titers in OF and serum at age 6,14 and 18weeks. For this analysis a cutoff will not be used. Instead, the simple correlation (represented by the R-squared value) will be calculated between anti-rotavirus IgA titer in serum compared with anti-rotavirus IgA titer in oral fluid. This will give the best indication of how well oral fluid antibody represents antibody in serum.

- 1. **Role of Rotavirus Neutralizing activity in Breast Milk on the immunogenicity of Rotavirus vaccine:**

The distribution of the level of rotavirus neutralizing activity in breast milk samples (among those infants who were rotavirus IgA negative at age 6 weeks and completed the study ATP) will be examined. The independent effect of the level of breast milk neutralizing activity on the rate of rotavirus IgA seroconversion at age 18 weeks will be assessed using logistic regression. Breast milk arm (immediate vs. withholding) and level of rotavirus neutralizing antibody in 6-week serum sample will be included as independent variables and effect modification will be examined. Analyses will also be performed using rotavirus IgA seroconversion rate at age 14 weeks as the endpoint. Exploratory analyses will be performed for correlation between level of breast milk neutralizing activity (in breast milk samples at ages, 6, 10 and 14 weeks) and rotavirus IgA level at ages 14 and 18 weeks in the different two arms.

- 1. . **Enrollment Time Period**

Refer to section 5

- 1. **Inclusion Criteria :**

Same as section 3.4

AND

- Mother reports she intends to breastfeed her infant through at least age 14 weeks (Exclusive or non exclusive breast feeding).
  1. **Exclusion Criteria**

Refer to section 3.5

AND

- Biological mother not available for participation in the study.
  1. **Elimination Criteria During the Study**

Refer to section 3.6

- 1. **Contraindication of the Vaccine**

Refer to section 3.7

**Study C: To evaluate baseline seroconversion rate for rotavirus among children in the first 18 weeks of life.**

- 1. **Study Site**

1. This study will be conducted at areas adjacent to Ali Akber Shah Goth and Ibrahim Haidry, which are currently not part of Department of Paediatrics and Child Health regular demographic surveillance area. Long term surveillance with provision of routine health services, similar to both study areas will be added to these sites.


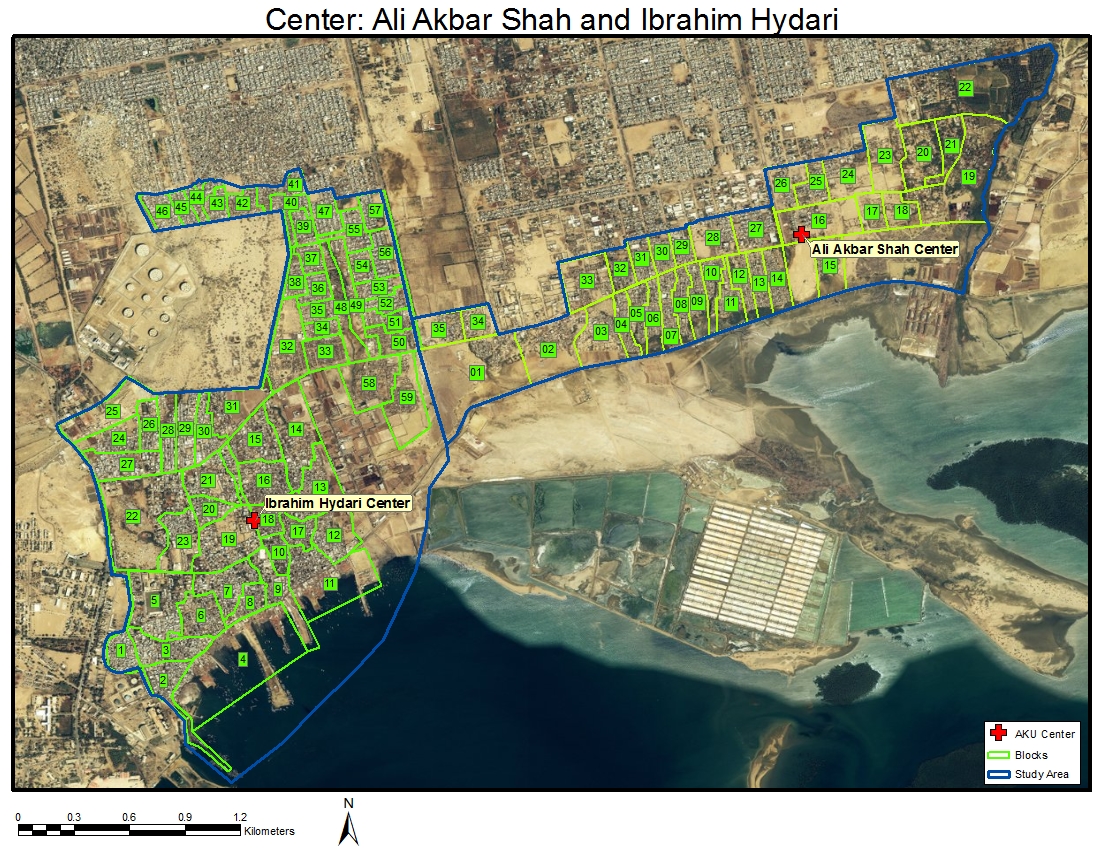


- 1. **Study Design**

This will be a baseline surveillance study extended in two specific areas adjacent to Ali Akber Shah Goth and Ibrahim Haidry. Children will receive the routine EPI vaccines according to the usual schedule in Pakistan at 6, 10 and 14 weeks of age at the PHC. Transportation will be provided for each visit. Refer to study schema section 7.17 for design of the study.

- 1. **Sample Size Calculations**

Target enrollment would be 100 infants. This group will provide the background rotavirus seroconversion. Estimate of background seroconversion from different parts of the world have ranged from <5% to up to 20%. Assuming the conservative seroconversion rate of 20% in baseline population and 40% in vaccinated arms, power at 0.8 and alpha error at 0.05, we will need 91 subjects in each arm to detect differences in IgA seroconversion in vaccinated and control arms. We plan to recruit 100 subjects in this group to provide this background rotavirus seroconversion rate.

- 1. **Inclusion Criteria**
  - 6 weeks 0 days to7 weeks 6 days age at the time of enrollment.
  - Healthy infant free of chronic or serious medical condition as determined by history and physical exam at time of enrollment into in the study.
  - Written informed consent obtained from the parents or guardians.
  1. **Exclusion Criteria**
- Use of any investigational drug or vaccine during the study period.
- *Use of any immunosuppressive drugs.
- *Enrolment in any other trial (Simplified Antibiotic Therapy for Neonatal Sepsis Trial, Management of Omphalitis Trial).
- *Immunoglobulin and/or blood products use since birth or during the study period.

- 1. **Elimination Criteria During the Study**
- None
  1. **Recruitment Process**

Recruitment will be conducted weekly, following the same recruitment process as described in section 3.8

- 1. **Study schema**

CHWs will recruit healthy infants between 4- 6 weeks of age on their routine surveillance

CHW will do the assessment of the infant through enrollment check list

Parents or caretaker of eligible infants will be provided transportation for required visits to PHC

Primary Health Care Center

Medical Officer

History and PE to assess eligibility

**Eligible Not eligible**

Consent requested by study

Physician

Exit

**Consent declined**

**Consent given**

Study Participation

- 1. **Vaccination and Blood Draw Schedule.** Blood samples will be obtained at the scheduled visits just prior to the administration of vaccines.

EPI Vaccination

n= 100

**Visit at 6 weeks**

- Pre vaccine blood draw
- EPI vaccination

**Visit at 10 weeks**

- EPI vaccination

**Visit at 14 weeks**

- EPI vaccination

**Visit at 18 weeks**

- Blood draw
  1. **Enrollment Time Period**

Same as 3.11 Refer to section 4.5

- 1. **Enrollment Schedule**

| ***Number of Subjects*** | **Youngest (Y): 6wk, 0dy**  **Oldest(O): 7wk, 6dy** | **Y: 10wk, 0dy**  **O: 12wk, 6dy** | **Y: 14wk, 0dy**  **O: 17wk 6dy** |
| --- | --- | --- | --- |
| 100 | OPV + DPT-Hep-B-Hib | OPV +  DPT-Hep-B-Hib | OPV +  DPT-Hep-B-Hib |

*Standard EPI in Pakistan is OPV plus BCG at birth; DPT-Hep-B-Hib and OPV at 6, 10, and 14 weeks, and measles at 9 months and second year of life

- 1. **Sampling Collection and Handling**

Same as 3.13

- 1. **Lab Assay**

| ***6wks*** | ***18 wks*** |
| --- | --- |
| Rotavirus IgA (Serum)  Rotavirus Neutralizing Antibody (Serum) | Rotavirus IgA (Serum) |

- 1. **Laboratory Training for Rotavirus antibody assays**

Same as 3.15

- 1. **Data Entry, Cleaning and Management**

Same as 3.16

- 1. **Data Analysis of Demographics**

Same as 3.17

- 1. **Analysis of immunogenicity**

Rotavirus IgA seroconversion rate by age 18 weeks (and 95% CI) will be determined for infants in each arm who were rotavirus IgA –negative at 6 weeks of age, using the same cut offs as described in section 3.18. The primary endpoint will be the rotavirus IgA seroconversion rate at age 18 weeks.

1. **HUMAN SUBJECT CONSIDERATION**
   1. **Ethics and Regulation Consideration**

The study would be conducted according to the guidelines for Good Clinical Practice in clinical trials, the declaration of Helsinki and local rules and regulation. Although Rotarix**®** vaccine is licensed in Pakistan, it is not currently recommended in Pakistan’s EPI. Determining the baseline seroconversion rate of rotavirus in the non-vaccinated population is important to determine the true impact of rotavirus vaccination. Although the population in the control group will not be getting rotavirus vaccine, we will provide extensive surveillance and counseling to this population through our active surveillance program. We will ensure that these children get all the routine EPI vaccines and transportation to the EPI centers will be provided to them. Free treatment for routine illnesses, according to the local standards, will be provided to them at our PHC center.

- 1. **Institutional Review Board**

Study protocol and associated study instruments, including consent forms in English and local language will be submitted to the Aga Khan University’s Ethical Review Committee for approval before commencement of any study activities. Same documents will be submitted to the PATH IRB for approval.

The study team, including doctors, paramedics and community health workers will be trained in human subject protection.

- 1. **Patient Data Confidentiality**

All the research documents and specimens will be held confidential and only shared with individuals who are directly involved in the study. Study questionnaire and data forms will be kept in a secured place and accessible only to study staff. Participant's information will be given a study code, and the data would be entered using these codes. The patient identifiers will be separated from the coded questionnaires and kept in locked file at study site or AKU. No personal identifiers will be entered in the data base. Data confidentiality will be maintained at all times. No personal identifiers will be used in any reports or publications of study.

- 1. **Informed Consent**

**Separate consent forms will be administered for each study component**

Verbal explanation regarding study will be given to all the parents of the subjects and a written consent form informing them of the nature of the study, its rationale, and its risks and benefits will be signed by parents of all subjects with appropriate witness signatures.

The informed consent in English will be translated into Urdu and back translated into English to ensure correct translation which will be verified by an independent (non-study) person. In case the parent or caretaker is illiterate, a thumb impression would be requested, with a witness signature.

Children whose families do not wish to participate, or those who want to withdraw will continue to receive all routine care and EPI vaccines that is offered free of charge at the PHC. There will be no negative consequences to health care services offered to these children at PHC. Patients who dropped out during the study would be asked if there previously collected data could be used in the analysis or not.

- 1. **Disseminating the Result to the public**

Results will be shared with the partners at CDC and PATH. Results will also be disseminated to pediatric and public health communities in Pakistan through scientific meetings and may be submitted for publication in a peer-reviewed journal with an international public health audience. While we cannot arrange for rotavirus vaccine to all children in Pakistan by ourselves, we will share our results with the policy makers of Pakistan’s EPI program and international health agencies for their action.

- 1. **Study Benefits**

We will ensure that the child gets complete EPI vaccination for the entire study period. All children will undergo active surveillance and free routine health care will be provided at the AKU run primary health care center in the area. Children/guardian will also receive a small non coercive gift pack containing (diaper, soap, shampoo, baby lotion) as compensation for their time in this study.

1. **Time line**

| **Year** | **2009** | | | | | | **2010** | | | | | | | | | | | | **2011** | | | | | | | | | | | | **2012** | | | |  | |  | |  |
| --- | --- | --- | --- | --- | --- | --- | --- | --- | --- | --- | --- | --- | --- | --- | --- | --- | --- | --- | --- | --- | --- | --- | --- | --- | --- | --- | --- | --- | --- | --- | --- | --- | --- | --- | --- | --- | --- | --- | --- |
| **Month** | **7** | **8** | **9** | **10** | **11** | **12** | **1** | **2** | **3** | **4** | **5** | **6** | **7** | **8** | **9** | **10** | **11** | **12** | **1** | **2** | **3** | **4** | **5** | **6** | **7** | **8** | **9** | **10** | **11** | **12** | **1** | **2** | **3** | **4** | | **5** | | **6** | |
| **Protocol Development** |  |  |  |  |  |  |  |  |  |  |  |  |  |  |  |  |  |  |  |  |  |  |  |  |  |  |  |  |  |  |  |  |  |  | |  | |  | |
| **Ethical Review Submission** |  |  |  |  |  |  |  |  |  |  |  |  |  |  |  |  |  |  |  |  |  |  |  |  |  |  |  |  |  |  |  |  |  |  | |  | |  | |
| **Contract Signing** |  |  |  |  |  |  |  |  |  |  |  |  |  |  |  |  |  |  |  |  |  |  |  |  |  |  |  |  |  |  |  |  |  |  | |  | |  | |
| **Recruitment & Training** |  |  |  |  |  |  |  |  |  |  |  |  |  |  |  |  |  |  |  |  |  |  |  |  |  |  |  |  |  |  |  |  |  |  | |  | |  | |
| **Standard Operating Procedures** |  |  |  |  |  |  |  |  |  |  |  |  |  |  |  |  |  |  |  |  |  |  |  |  |  |  |  |  |  |  |  |  |  |  | |  | |  | |
| **Field Pilot Testing** |  |  |  |  |  |  |  |  |  |  |  |  |  |  |  |  |  |  |  |  |  |  |  |  |  |  |  |  |  |  |  |  |  |  | |  | |  | |
| **Subject Recruitment for component A, B & C** |  |  |  |  |  |  |  |  |  |  |  |  |  |  |  |  |  |  |  |  |  |  |  |  |  |  |  |  |  |  |  |  |  |  | |  | |  | |
| **Data Analysis & Report Writing** |  |  |  |  |  |  |  |  |  |  |  |  |  |  |  |  |  |  |  |  |  |  |  |  |  |  |  |  |  |  |  |  |  |  | |  | |  | |

**Reference List**

(1) Generic protocol for monitoring impact of rotavirus vaccination on gastroenteritis disease burden and viral strains. 2009. World Health Organization Department of Immunization, Vaccines and Biologicals CH-1211 Geneva 27, Switzerland. Ref Type: Generic

(2) Glass RI, Bresee J, Jiang B, Parashar U, Yee E, Gentsch J. Rotavirus and rotavirus vaccines. Adv Exp Med Biol 2006; 582:45-54.

(3) Parashar UD, Gibson CJ, Bresse JS, Glass RI. Rotavirus and severe childhood diarrhea. Emerg Infect Dis 2006 February;12(2):304-6.

(4) Parashar UD, Hummelman EG, Bresee JS, Miller MA, Glass RI. Global illness and deaths caused by rotavirus disease in children. Emerg Infect Dis 2003 May;9(5):565-72.

(5) Glass RI, Parashar UD, Bresee JS, Turcios R, Fischer TK, Widdowson MA et al. Rotavirus vaccines: current prospects and future challenges. Lancet 2006 July 22;368(9532):323-32.

(6) Ruiz-Palacios GM, Perez-Schael I, Velazquez FR, Abate H, Breuer T, Clemens SC et al. Safety and efficacy of an attenuated vaccine against severe rotavirus gastroenteritis. N Engl J Med 2006 January 5; 354(1):11-22.

(7) Vesikari T, Matson DO, Dennehy P, Van DP, Santosham M, Rodriguez Z et al. Safety and efficacy of a pentavalent human-bovine (WC3) reassortant rotavirus vaccine. N Engl J Med 2006 January 5; 354(1):23-33.

(8) Meeting of the Immunization Strategic Advisory Group of Experts, April 2009 - conclusions and recommendations. Weekly epidemiological record 23[84], 213-236. 6-5-2009. World Health Organization.
Ref Type: Generic

(9) Meeting of the immunization
Strategic Advisory Group of Experts,
April 2009 – conclusions and
recommendations. Weekly epidemiological record 23[84], 213-236. 6-5-2009. World Health Organization.
Ref Type: Generic

(10) Meeting of the immunization
Strategic Advisory Group of Experts,
April 2009 – conclusions and
recommendations. Weekly epidemiological record 23[84], 213-236. 6-5-2009. World Health Organization.
Ref Type: Generic

(11) Steele AD, De VB, Tumbo J, Reynders J, Scholtz F, Bos P et al. Co-administration study in South African infants of a live-attenuated oral human rotavirus vaccine (RIX4414) and poliovirus vaccines. Vaccine 2008 September 8.

(12) Patel M, Shane A, Parashar U, Jiang B, Gentsch J, Glass R. **Oral Rotavirus Vaccines: How well will they work where they are needed most?** J Infect Dis. 2009 Nov 1;200 Suppl 1:S39-48. Review

(13) Newburg DS, Peterson JA, Ruiz-Palacios GM, Matson DO, Morrow AL, Shults J et al. Role of human-milk lactadherin in protection against symptomatic rotavirus infection. Lancet 1998 April 18; 351(9110):1160-4.

(14) Rennels MB. Influence of breast-feeding and oral poliovirus vaccine on the immunogenicity and efficacy of rotavirus vaccines. J Infect Dis 1996 September;174 Suppl 1:S107-S111.

(15) Patel M, Shane A, Parashar U, Jiang B, Gentsch J, Glass R. **Oral Rotavirus Vaccines: How well will they work where they are needed most?** Journal of Infectious Diseases . 2009.
Ref Type: Generic
