# Supplementary material for: Impact of Withholding Breastfeeding at the Time of Vaccination on the Immunogenicity of Oral Rotavirus Vaccine—A Randomized Trial
Source: PLoS One. 2015 Jun 2;10(6):e0127622. doi: 10.1371/journal.pone.0127622 (PMC4452702; doi:10.1371/journal.pone.0127622)
Supplement: S1 Related Publication — (PDF) [file pone.0127622.s004.pdf]

Journal of Infectious Diseases Advance Access published July 7, 2014

MAJOR ARTICLE

Impact of Different Dosing Schedules on the Immunogenicity of the Human Rotavirus Vaccine in Infants in Pakistan: A Randomized Trial

Syed Asad Ali,[1] Abdul Momin Kazi,[1] Margaret M. Cortese,[2] Jessica A. Fleming,[3] Umesh D. Parashar,[2] Baoming Jiang,[2] Monica Malone McNeal,[4] Duncan Steele,[3,5] Zulfiqar Bhutta,[1] and Anita Zaidi[1]

[1]Department of Pediatrics and Child Health, Aga Khan University, Karachi, Pakistan; [2]Division of Viral Diseases, National Center for Immunization and Respiratory Diseases, Centers for Disease Control and Prevention, Atlanta, Georgia; [3]Vaccine Access and Delivery, Program for Appropriate Technology in Health (PATH), Seattle, Washington; [4]Department of Pediatrics, Division of Infectious Diseases, Cincinnati Children's Hospital Medical Center, Cincinnati, Ohio; and [5]Now With The Bill & Melinda Gates Foundation, Seattle, Washington

Background. Current oral rotavirus vaccines perform suboptimally in resource-poor settings. We investigated the effect of an additional dose and later schedule on the immunogenicity of monovalent rotavirus vaccine (RV1) in a developing country.

Methods. Infants received RV1 at 6 and 10, 10 and 14, or 6, 10, and 14 weeks of age. The primary objective was to compare antirotavirus immunoglobulin A (IgA) seroconversion at 18 weeks in the 6/10/14 arm to the cumulative seroconversion (highest result at 14 or 18 weeks) in the 6/10 arm.

Results. Overall, 480 (76.2%) of 630 randomized infants completed the trial per protocol. Seroconversion in the 6/10/14 arm was 36.7% (95% CI, 29.8, 44.2) compared to 36.1% (CI, 29.0, 43.9) in the 6/10 arm, ($P = 1.0$); the result from the 10/14 arm was 38.5% (CI, 31.2, 46.3). Seroconversion in the 6/10 arm at 14 weeks (post hoc) was lower at 29.7% (CI, 23.1, 37.3).

Conclusions. In Pakistani infants, the immunogenicity of RV1 did not increase significantly with 3 doses at 6, 10, and 14 weeks compared to 2 doses at 6 and 10 weeks. Additional strategies should be evaluated for improving rotavirus vaccine immunogenicity in high burden countries.

Keywords. rotavirus; rotavirus vaccine; vaccination schedule; Rotarix; Pakistan.

Rotavirus is the most common cause of severe gastroenteritis in children <5 years of age, accounting for an estimated 453 000 deaths in 2008 [1]. Around 85% of these deaths occur in the developing countries of Asia and Africa. In Pakistan, rotavirus causes 5.5 episodes of moderate to severe diarrhea per 100 infant-years, accounting for 23% of all moderate to severe diarrhea in infants in the country [2]. Because of the tremendous global burden, rotavirus vaccine development and introduction has been a high global priority. In 2006, 2 oral rotavirus vaccines were licensed for use in many countries: 3-dose RotaTeq (Merck and Co, Inc, Whitehouse Station, NJ) (RV5) and 2-dose Rotarix (GlaxoSmithKline Biologicals, Rixensart, Belgium) (RV1). The pivotal trials of both these vaccines, conducted mainly in high- and middle-income countries, demonstrated high efficacy (85%–98%) against severe rotavirus disease and a good safety profile [3, 4]. Subsequent evaluations in middle- and low-income countries demonstrated that, as with other oral vaccines, immunogenicity and efficacy were lower, with efficacy ranging from 30% to 80% [5–9].

The reasons for lower immunogenicity and efficacy of oral rotavirus vaccines in low-income settings are not completely understood but may be related to a

Received 24 March 2014; accepted 21 May 2014.
Correspondence: Syed Asad Ali, MBBS, MPH, Assistant Professor, Department of Paediatrics and Child Health, Aga Khan University, Stadium Road, Karachi, Pakistan (asad.ali@aku.edu).

The Journal of Infectious Diseases
© The Author 2014. Published by Oxford University Press on behalf of the Infectious Diseases Society of America. All rights reserved. For Permissions, please e-mail: journals.permissions@oup.com.
DOI: 10.1093/infdis/jiu335

Downloaded from http://jid.oxfordjournals.org/ at Aga Khan University on January 2, 2015

number of factors, including higher titers of maternal antibodies in infants, interference by oral polio vaccines (OPV), micronutrient deficiencies, concomitant gut infection with other pathogens, environmental enteropathy, altered gut microbiome, and concomitant host comorbidities such as diarrhea. In low-income settings, younger ages at doses generally yield lower rotavirus vaccine responses. For example, an immunogenicity study in South Africa using a lower-titer precursor to RV1 demonstrated that infants given 2 doses of vaccine at ages 6 and 10 weeks had reduced immune response compared to that in infants who received 2 doses at 10 and 14 weeks (antirotavirus immunoglobulin A [IgA] seroconversion 36% vs 61%, respectively) [10]; these schedules have not been compared using the licensed RV1 titer. In a secondary analysis of a trial in South Africa and Malawi that evaluated 2 different RV1 schedules, the overall efficacy during the follow-up period to 1 year of age of a 2-dose RV1 schedule at 10 and 14 weeks (59%) was not statistically different from that following a 3-dose schedule at 6, 10, and 14 weeks (64%), suggesting that a first dose given at 6 weeks did not provide notable additional benefit to the later 2-dose schedule; however, it did appear to be beneficial through the second year of life in a follow-up analysis in South Africa (2- and 3-dose efficacy: 32% vs 85%, respectively) [6].

The immunogenicity and efficacy of a 2-dose RV1 schedule at 6 and 10 weeks has not been assessed in a clinical trial, but the World Health Organization (WHO) recommends RV1 be given at the first and second routine Expanded Program on Immunization (EPI) encounters, which is at ages 6 and 10 weeks in most low-income countries. Because an additional dose at 14 weeks (third EPI visit) or a later administration of the 2 doses would allow for less potential interference with maternal antibodies and further maturation of the immune system, we conducted a randomized trial to compare the immunogenicity of RV1 when administered in a 3-dose schedule at 6, 10, and 14 weeks to the routine schedule of 6 and 10 weeks (primary objective), and to compare the 6- and 10-week schedule with a later 2-dose schedule at 10 and 14 weeks. We also evaluated the correlation of maternally derived neutralizing antibody (MDNA) levels with antirotavirus IgA antibody seroconversion.

METHODS

Study Participants

The study was conducted in the peri-urban area of Ali Akber Shah Goth, in Karachi, Pakistan, where the Department of Pediatrics and Child Health of Aga Khan University has conducted demographic surveillance since 2008, including pregnancy and newborn surveillance. It is a low-income community of predominantly fishermen, located along the sea coast. Among neonates and children <5 years of age, mortality figures in this population are 43.4 and 66.4 per 1000 live births, respectively.

Parents were informed of the study when their infants were 4 weeks of age, and if they gave consent, their infants were enrolled at 6 weeks of age. Infants were excluded if they had a birth weight <1500 grams, received rotavirus vaccine outside of the study, had received any immunoglobulin or blood products since birth, were on immunosuppressive drugs, or had used any investigational drug or vaccine within 30 days of the first dose of study vaccine.

The trial was approved by the institutional review boards of Aga Khan University, the United States Centers for Disease Control and Prevention, and the Western Institutional Review Board (Olympia, WA). The study was conducted in accordance with the principles of the Declaration of Helsinki and in compliance with good clinical practice guidelines (ClinicalTrials.gov registration number NCT01199874).

Study Design

This Phase IV, open-label, randomized trial of 630 children included 3 parallel intervention arms with treatment allocation of 1:1:1. One group received 3 doses of RV1 at 6, 10, and 14 weeks of age (6/10/14 arm), the second group received 2 doses of RV1 at 6 and 10 weeks of age (6/10 arm), and the third group received 2 doses of RV1 at 10 and 14 weeks (10/14 arm). The lyophilized formulation of RV1 was provided along with oral polio vaccine (OPV) and pentavalent vaccine (diphtheria, tetanus, whole cell pertussis, hepatitis B, and *Haemophilus influenzae* type b), unless either had been administered earlier at another facility. Vaccinations were postponed if the child was suffering from fever ≥38.0°C, had vomited during each of the last 3 feedings, or met WHO criteria for some or severe dehydration [11]. Feeding was not restricted before or after vaccination. To provide an estimate of the background antirotavirus IgA seroconversion, 110 infants from a neighboring area were enrolled at age 6 weeks and thereafter received EPI vaccines but no rotavirus vaccine ("comparison group"); a similar proportion of infants in this group and the intervention arms were enrolled during each calendar week to maintain comparability of the likelihood of exposure to wild-type rotavirus during the study period. The study schedule is summarized in Table 1.

Laboratory Assays

Antirotavirus IgA antibody was measured in serum collected at age 6 weeks (prevaccination) and 18 weeks in all infants; infants in the 6/10 arm had additional serum collected at 14 weeks. The concentration of antirotavirus IgA antibody in serum was measured using an enzyme-linked immunosorbent assay (ELISA) as described elsewhere [12, 13], expressed as units per milliliter (U/mL). Preexisting neutralizing antibodies, presumed to be maternally derived (MDNA), were measured in serum collected at 6 weeks by a neutralization assay against rotavirus strain 89-12, the precursor to the RV1 vaccine strain, following methods

Downloaded from http://jid.oxfordjournals.org/ at Aga Khan University on January 2, 2015

Table 1. Vaccination and Specimen Collection Schedule

	Study Visit 1 (V1) 6 wks	Study Visit 2 (V2) 10 wks	Study Visit 3 (V3) 14 wks	Study Visit 4 (V4) 18 wks
Study Arm	Age: 42–55 d	Age: 25 to 41 d post V1 (6/10, 6/10/14 arms); 70–83 d (10/14 arm)	Age: 25–41 d post V2	Age: 25–41 d post V3
6/10	Eligibility assessment<br>Baseline assessment<br>Blood draw<br>Rotavirus and EPI vaccination	Clinical assessment<br>Rotavirus and EPI vaccination	Clinical assessment<br>Blood draw<br>EPI vaccination	Clinical assessment<br>Blood draw<br>Exit from study
10/14	Eligibility assessment<br>Baseline assessment<br>Blood draw<br>EPI vaccination	Clinical assessment<br>Rotavirus and EPI vaccination	Clinical assessment<br>Rotavirus and EPI vaccination	Clinical assessment<br>Blood draw<br>Exit from study
6/10/14	Eligibility assessment<br>Baseline assessment<br>Blood draw<br>Rotavirus and EPI vaccination	Clinical assessment<br>Rotavirus and EPI vaccination	Clinical assessment<br>Rotavirus and EPI vaccination	Clinical assessment<br>Blood draw<br>Exit from study
Comparison	Eligibility assessment<br>Baseline assessment<br>Blood draw<br>EPI vaccination	Clinical assessment<br>EPI vaccination	Clinical assessment<br>EPI vaccination	Clinical assessment<br>Blood draw<br>Exit from study

Abbreviation: EPI, Expanded Program on Immunization.

described elsewhere [12, 13]. Data are expressed as serum titers that represent 60% neutralization of the virus.

Safety Assessment

All infants were observed for 30 minutes after the administration of vaccines, and parents were asked to seek care at the study clinic for any illnesses in participants during the study period. All serious adverse events (SAEs) were reported to an independent safety monitor who characterized possible relationship to study product.

Statistical Analysis

Analyses were conducted on the per protocol cohort, which included infants meeting all inclusion and no exclusion criteria who were anti-rotavirus IgA seronegative at 6 weeks, received RV1 vaccines and blood collection according to schedule, and remained in the study through 18 weeks. Seroconversion was defined as a serum concentration of antirotavirus IgA antibodies ≥20 U/mL in subjects who were seronegative (IgA <20 U/mL) at 6 weeks of age. For the 6/10/14 and the 10/14 arms, seroconversion was determined at 18 weeks, approximately 4 weeks after the last RV1 dose; for the 6/10 arm, it was based on the higher IgA titer of each subject's 14 or 18 week sample. In this setting where rotavirus circulation occurs throughout the year, assessing IgA at both time points for the 6/10 arm allowed infants in all intervention arms to have comparable lengths of time for possible infection from wild-type rotavirus (at 18 weeks) and also comparable lengths of time for possible waning of vaccine-induced IgA (4 weeks after the last vaccine dose). In post hoc analyses, seroconversion in the 6/10 arm independently at 14 weeks and 18 weeks was compared with seroconversion at 18 weeks in the 10/14 and 6/10/14 arms.

Rotavirus IgA seroconversion between the 6/10 arm and the 6/10/14 and 10/14 arms were compared using the Fisher exact test, 95% confidence intervals (CI) of seroconversion were based on the Wilson Score method, and 95% CI of the differences in seroconversion were calculated based on the Newcombe-Wilson method without continuity correction [14]. Serum anti-rotavirus IgA geometric mean titers (GMT) were compared across arms using the *t* test for all infants (a value of 10U.mL was assigned for infants with a IgA level of <20 U/mL), as well as using only infants who had seroconverted.

The GMTs of the 6-week MDNA titers were compared across intervention arms using the *t* test. To examine the impact of MDNA on seroconversion, the 6-week MDNA titers were pooled and quartiles determined. Within each arm, seroconversion for infants with MDNA titers in the lowest quarter (≤25th percentile) was compared with that for infants in the highest quarter (≥75th percentile) using the Fisher exact test. Data analysis was performed using SAS software (SAS Institute Inc, North Carolina).

Enrollment of 200 infants in each intervention arm was estimated to provide 85% power to detect a 17.5% increase in seroconversion in the 6/10/14 arm compared to the 6/10 week arm,

Downloaded from http://jid.oxfordjournals.org/ at Aga Khan University on January 2, 2015

using a 2-sided χ^2 test ($\alpha = 0.05$), assuming 35% seroconversion in the 6/10 arm, 5% IgA seropositivity at 6 weeks, and a 20% dropout during the study. In the comparison group, 110 subjects were recruited for determining background antirotavirus IgA antibody seroconversion between ages 6 and 18 weeks.

RESULTS

Study Population

The study was conducted from April 2011 to September 2012. Of the 624 infants in the intervention arms with serum evaluated at 6 weeks, 52 (8.3%) were antirotavirus IgA positive at 6 weeks of age and excluded from the immunogenicity analyses (Figure 1). Overall, 480 of 630 enrolled (76.2%) infants completed the study according to the protocol. Arms were similar with respect to age, gender, length, weight, and family/household characteristics at the time of enrollment (Table 2). The mean age of infants at each study visit was similar across intervention arms and the comparison group. OPV was given concomitantly with each dose of RV1 in ≥99% of participants in each of the intervention arms; the remaining children had documentation of receiving OPV doses from an outside facility. Birth dose of OPV was given to 75% of the infants, and this distribution was similar across all arms. Additional bOPV/mOPV doses were likely received by the study infants during campaigns that occurred during the trial.

Immune Response to RV1

Antirotavirus IgA antibody seroconversion was 36.7% (95% CI, 29.8, 44.2) in the 6/10/14 arm, 36.1% (95% CI, 29.0, 43.9) in the 6/10 arm (cumulative result, by age 18 weeks), and 38.5% (95% CI, 31.2, 46.3) in the 10/14 arm (difference: 6/10/14 arm vs 6/10 arm, 0.6% [95% CI, −9.9, 10.9]; 10/14 arm vs 6/10 arm, 2.3% [95% CI, −8.3, 12.9]) (Table 3). Background seroconversion in the comparison group was 13.3% (95% CI, 7.8, 21.9) at age 18 weeks. Antirotavirus IgA GMT postvaccination was 25.8 U/mL in the 6/10/14 arm, 24.0 U/mL in the 6/10 arm (cumulative result, by age 18 weeks), and 24.4 U/mL in the 10/14 arm. The GMT in the comparison group was 14.4 U/mL at 18 weeks of age. The GMTs of infants who seroconverted postvaccination were similar across intervention arms (Table 4).

In post-hoc analyses, seroconversion in the 6/10 arm was 29.7% and 28.4% at 14 weeks and 18 weeks, respectively (difference: 6/10/14 arm at 18 weeks vs 6/10 arm at 14 weeks: 7.0% [95% CI, −3.3, 17.0]; 10/14 arm at 18 weeks vs 6/10 arm at 14 weeks: 8.8% [95% CI, −1.7, 19.0] (Table 3). In the 6/10 arm, 12/46 (26.1%) IgA seropositive infants at 14 weeks (4 weeks after the last RV1 dose) were seronegative at 18 weeks, whereas 10/

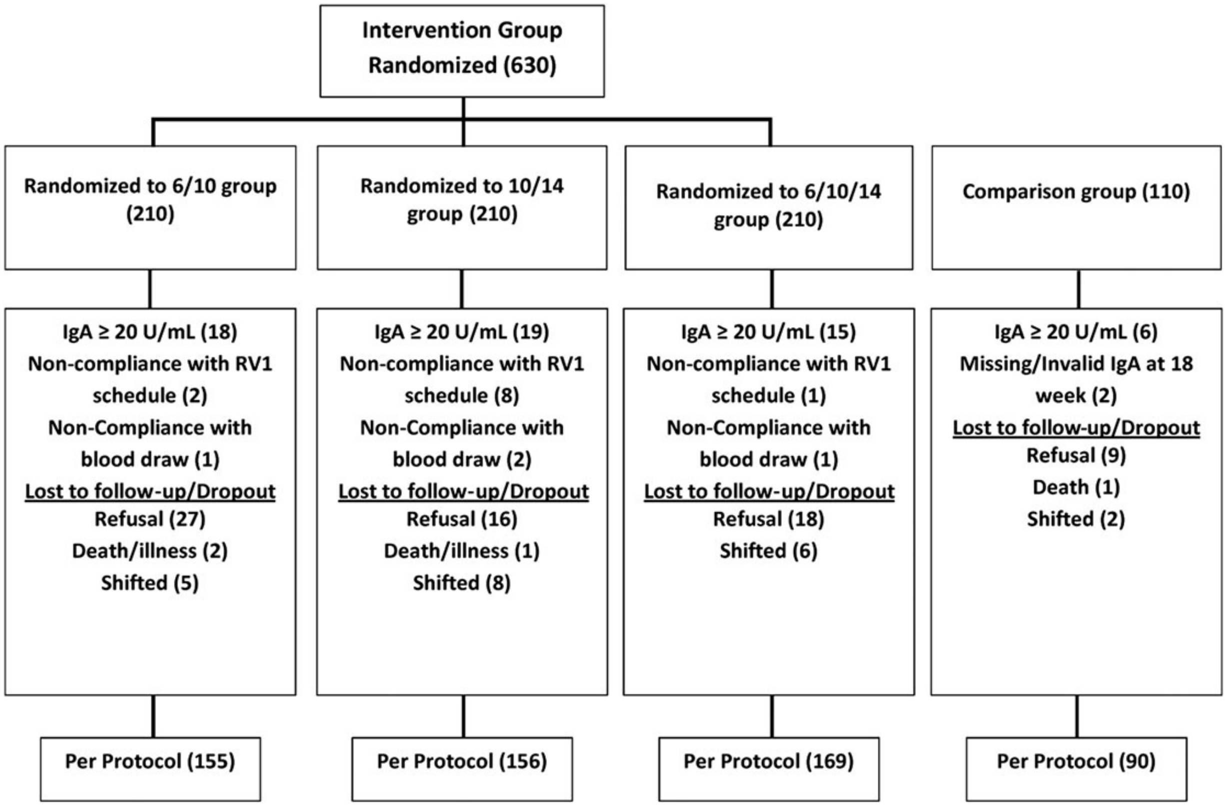

Figure 1. Study assignment and follow-up of subjects.

Downloaded from http://jid.oxfordjournals.org/ at Aga Khan University on January 2, 2015

Table 2. Baseline Characteristics and Age at Study Visit

	6/10 Arm (N = 155)		10/14 Arm (N = 156)		6/10/14 Arm (N = 169)		Comparison Arm (N = 90)	
	N	% or Mean	n	% or Mean	n	% or Mean	n	% or Mean
Baseline Enrollment Characteristics								
Age, weeks (SD)	155	6.4 (0.5)	156	6.3 (0.4)	169	6.3 (0.5)	90	6.4 (0.5)
Male	80	51.9	81	51.9	85	50.6	47	52.2
Length at 6 weeks, cm (SD)	154	52.8 (3.3)	156	53.1 (2.9)	169	52.6 (3.6)	90	53.7 (2.7)
Weight at 6 weeks, kg (SD)	154	4.06 (0.66)	156	4.16 (0.67)	169	4.07 (0.64)	90	4.07 (0.64)
FOC at 6 weeks, cm (SD)	154	36.9 (1.9)	156	36.7 (1.5)	169	36.6 (1.2)	90	36.7 (1.8)
Age of mother, years (SD)	155	24.9 (4.8)	156	25.5 (4.7)	169	26.5 (5.2)	90	24.7 (4.4)
Mother years of schooling (SD)	155	5.1 (4.5)	156	5.0 (4.4)	169	4.5 (4.3)	90	3.1 (4.1)
Father years of schooling (SD)	155	5.0 (4.6)	155	4.8 (4.7)	168	4.9 (4.5)	90	4.3 (5.0)
Type of milk								
Breast milk only	116	74.8	128	82.1	131	77.5	78	86.7
Breast milk plus formula	29	18.7	25	16.0	32	18.9	11	12.2
Formula only	10	6.5	3	1.9	6	3.6	1	1.1
Main water source								
Piped	135	87.1	121	77.6	144	85.2	61	67.8
Tube/deep tube well	1	0.6	0	0.0	2	1.2	2	2.2
Surface water	7	4.5	22	14.1	8	4.7	16	17.8
Bottled/filtered	0	0.0	1	0.6	2	1.2	8	8.9
Other	12	7.7	12	7.7	13	7.7	3	3.3
Main roof material								
Jute/bamboo/mud	4	2.6	5	3.2	2	1.2	2	2.2
Tin	23	14.8	25	16.0	21	12.4	13	14.4
Cement/concrete	128	82.6	126	80.8	146	86.4	75	83.3
Main toilet type								
Septic tank/modern toilet	9	5.8	13	8.3	17	10.1	8	8.9
Pit latrine, water sealed	145	93.5	142	91.0	149	88.2	54	60.0
Pit latrine, not water sealed	1	0.6	1	0.6	3	1.8	28	31.1
Place of delivery								
Home	55	35.7	53	34.0	58	34.3	38	42.2
Hospital/Clinic	99	64.3	103	66.0	111	65.7	52	57.8
Age at Study Visit, weeks (SD)								
Visit 2	155	10.7 (0.7)	156	10.5 (0.5)	169	10.6 (0.6)	90	10.6 (0.6)
Visit 3	155	14.9 (0.8)	156	14.7 (0.7)	169	14.7 (0.7)	90	14.8 (0.8)
Visit 4	155	19.1 (0.9)	156	18.9 (0.8)	169	18.9 (0.8)	90	19.0 (1.0)

Abbreviations: FOC, Fronto-occipital circumference; SD, Standard deviation.

109 (9.2%) IgA seronegative infants at 14 weeks seroconverted by 18 weeks.

Impact of Maternally-Derived Neutralizing Antibody Level on Immunogenicity

The GMTs of the titers of MDNA against rotavirus measured in infants' serum at age 6 weeks were similar in the intervention arms; 113.6 U/mL (95% CI, 95.7, 134.8), 100.2 U/mL (95% CI, 83.9, 119.7), and 101.2 U/mL (95% CI, 84.9, 120.7), respectively, in the 6/10/14, 6/10, and 10/14 arms; the distributions of MDNA titers were also similar (data not shown). In the 6/10/14 arm, seroconversion (at 18 weeks) for infants with low MDNA titers (≤25th percentile, using MDNA titers pooled from all intervention arms) compared to that among infants with high MDNA titers (≥75th percentile) was 44.7% and 34.1%, respectively (Table 5). Seroconversion in the 6/10 arm (at 14 weeks, 4 weeks after last dose) for infants with low vs high MDNA titers was 31.6% and 19.5%, respectively; the 10/14 arm values at 18 weeks were 59.1% and 20.0%. Although within each arm seroconversion 4 weeks after the last dose tended to be greater among infants with the lowest MDNA titers compared to infants with highest MDNA titers, the difference reached statistical significance only in the 10/14 arm (P value = .001).

Downloaded from http://jid.oxfordjournals.org/ at Aga Khan University on January 2, 2015

Table 3. Percent Antirotavirus IgA Seroconversion and Difference Between Study Arms

	6/10 Arm (N = 155)			10/14 Arm (N = 156)			6/10/14 Arm (N = 169)			6/10/14 vs 6/10		10/14 vs 6/10		6/10/14 vs 10/14		Comparison Arm (N = 90)		
Sample	n	%	95% CI	n	%	95% CI	n	%	95% CI	Difference in Seroconversion % (95% CI)	*P* Value	Difference in Seroconversion % (95% CI)	*P* Value	Difference in Seroconversion % (95% CI)	*P* Value	n	%	95% CI
6 week	0	0		0	0		0	0								0	0	
14 week	46	29.7	(23.1, 37.3)							7.0 (−3.3, 17.0)	.20[a]	8.8 (−1.7, 19.0)	.12[a]					
18 week	44	28.4	(21.9, 35.9)	60	38.5	(31.2, 46.3)	62	36.7	(29.8, 44.2)	8.3 (−1.9, 18.2)	.12[b]	10.1 (−.4, 20.3)	.07[b]	−1.8 (−12.2, 8.7)	.82	12	13.3	(7.8, 21.9)
Cumulative 14 and 18 wks	56	36.1	(29.0, 43.9)							.6 (−9.9, 10.9)	1.00[c]	2.3 (−8.3, 12.9)	.73[c]					

Abbreviations: CI, confidence interval; IgA, immunoglobulin A.

[a] % seroconversion at 14 weeks in 6/10 arm compared with % seroconversion at 18 weeks in 10/14 or 6/10/14 arm.

[b] % seroconversion at 18 weeks in 6/10 arm compared with % seroconversion at 18 weeks in 10/14 or 6/10/14 arm.

[c] % seroconversion in 6/10 arm based on the higher IgA value among subjects' 14 and 18 weeks result vs % seroconversion at 18 weeks for subjects in 10/14 arm or 6/10/14 arm.

Table 4. Anti-Rotavirus IgA Geometric Mean Titers (GMT) (U/mL) and Comparisons Between Study Arms

	6/10 Arm			10/14 Arm			6/10/14 Arm			6/10/14 vs 6/10	10/14 vs 6/10	6/10/14 vs 10/14	Comparison Arm		
Sample	n	GMT	95% CI	n	GMT	95% CI	n	GMT	95% CI	*P* Value	*P* Value	*P* Value	n	GMT	95% CI
All subjects															
6 wk	155	10.0	(10.0, 10.0)	156	10.0	(10.0, 10.0)	169	10.0	(10.0, 10.0)				90	10.0	(10.0, 10.0)
14 wk	155	19.7	(16.2, 23.9)							.08[a]	.15[a]				
18 wk	155	18.4	(15.2, 22.2)	156	24.4	(19.5, 30.6)	169	25.8	(20.5, 32.5)	.02[b]	.06[b]	.74[b]	90	14.4	(11.5, 18.1)
Cumulative 14 and 18 wks	155	24.0	(19.2, 29.9)							.65[c]	.91[c]				
Seroconverted subjects															
6 wk	0	10.0	(10.0, 10.0)	0	10.0	(10.0, 10.0)	0	10.0	(10.0, 10.0)				0	10.0	(10.0, 10.0)
14 wk	46	98.1	(69.5, 138.4)							.24[a]	.88[a]				
18 wk	44	85.3	(50.8, 125.4)	60	101.9	(71.4, 145.4)	62	132.5	(92.5, 189.7)	.10[b]	.50[b]	.30[b]	12	154.8	(58.8, 408.0)
Cumulative 14 and 18 wks	56	112.3	(79.1, 159.4)							.51[c]	.70[c]				

Abbreviations: CI, confidence interval; GMT, geometric mean titer; IgA, immunoglobulin A.

[a] GMT at 14 weeks in 6/10 arm and at 18 weeks in 10/14 and 6/10/14 arms.

[b] GMT at 18 weeks in 6/10, 10/14, and 6/10/14 arms.

[c] GMT of higher IgA titer among subjects' 14 and 18 week results for 6/10 arm and at 18 weeks in 10/14 and 6/10/14 arms.

Downloaded from http://jid.oxfordjournals.org/ at Aga Khan University on January 2, 2015

Table 5. Comparison of Ranked MDNA Titers With Serum Antirotavirus IgA Seroconversion

	6/10 Arm (N = 154)		10/14 Arm (N = 155)		6/10/14 Arm (N = 169)	
	Seroconversion[a]		Seroconversion		Seroconversion	
MDNA Percentile	n/N	% (95% CI)	n/N	% (95% CI)	n/N	% (95% CI)
≤25th	12/38	31.6 (19.1, 47.5)	26/44	59.1 (44.4, 72.3)	17/38	44.7 (30.2, 60.3)
>25th and ≤50th	15/44	34.1 (21.9, 48.9)	12/34	35.3 (21.5, 52.1)	12/42	28.6 (17.2, 43.6)
>50th and <75th	11/32	34.4 (20.4, 51.7)	15/43	34.9 (22.4, 49.8)	18/45	40.0 (27.0, 54.6)
≥75th	8/41	19.5 (10.2, 34.0)	7/35	20.0 (10.0; 35.9)	15/44	34.1 (21.9, 48.9)
P value[b]	.30		.001		.37	

Abbreviations: CI, confidence interval; MDNA, maternally derived neutralizing antibodies.

[a] Seroconversion at 14 weeks in 6/10 arm, 4 weeks after last RV1 dose.

[b] *P* value comparing seroconversion in lowest vs highest MDNA quarters. (MDNA Q1 = 52.8, median = 107.1, Q3 = 231.6).

Safety Assessment

Two SAEs were observed in the 6/10/14 arm, 4 in the 6/10 arm, and 2 in the 10/14 arm; 1 SAE was identified in the comparison arm. An independent safety monitor found none of the SAEs related to the study vaccine.

DISCUSSION

In this study, administering RV1 in a 3-dose schedule at 6/10/14 weeks did not lead to significantly higher rotavirus IgA seroconversion at 18 weeks when compared to the cumulative seroconversion (highest IgA result at 14 or 18 weeks) following a 2-dose schedule at 6/10 weeks. Additionally, a delayed 2-dose schedule at 10/14 weeks did not lead to higher seroconversion compared to the cumulative result in the 6/10 group. Our study design whereby the 14 week and 18 week samples were cumulatively considered for the 6/10 arm seroconversion endpoint ultimately raised the bar for detecting statistically higher seroconversion in the other arms because a number of infants in the 6/10 arm changed seroconversion status between 14 and 18 weeks without an additional dose of RV1. In post hoc analyses, the 6/10/14 and 10/14 seroconversion point estimates at 18 weeks were higher (by 7 to 10 percentage points) than those of the 6/10 arm, when the 14 week and the 18 week results were considered separately, but the differences were not statistically significant. Within the strictly followed vaccination schedule of our infant series, the alternate schedules we tested did not greatly enhance the overall IgA response in our study population. Although the antirotavirus serum IgA antibody response is just one component of the immunological response to the vaccine and is not necessarily a mechanistic correlate of protection, a substantial improvement in clinical protection through these alternative vaccine schedules may not be likely in this population.

Our study area was a particularly poor slum setting where many of the risk factors for decreased immunogenicity of oral vaccines including malnutrition and environmental enteropathy were likely present, which may in part explain the failure to substantially improve the immune response by administering an additional third dose of RV1 at 14 weeks. The IgA seroconversion rates and overall GMTs for the intervention arms we describe here are among the lowest reported for RV1, even among studies performed in lower-income, high under-5 mortality countries and in which, like ours, OPV was coadministered with each RV1 dose [6, 10, 14–16]. In these studies, the IgA was measured by the laboratory used in our study or that of GSK; there may be differences in the 2 assays that could affect comparability of results.

When examining data across immunogenicity studies, the seroconversion in placebo or control arms should be considered to better compare the contribution of vaccine; seroconversion in placebo or control arms in previously cited evaluations in low-income countries ranged from 1.7% to 40.4%. In our study, there was 13.3% seroconversion between 6 and 18 weeks in the comparison group (and 9.2% in 6/10 week arm between 14 and 18 weeks), most likely due to natural infection or exposure. The results from our 6/10/14 and 10/14 arms (seroconversion 23 and 25 percentage points above comparison arm and overall GMT ratio of vaccine arm to comparison arm of 1.8 and 1.7, respectively) appear generally comparable to that reported in a follow-up analysis of the Malawi trial, where immunogenicity of 2-(mean age at vaccination, 11.7 and 16.8 weeks) and 3-doses of RV1 (mean age, 6.5, 11.7, and 16.8 weeks) was 47.2% and 57.1%, respectively, but with 40.5% seroconversion in the placebo arm (difference between vaccine arms and placebo 6.7% and 16.6%, respectively; ratio of GMT: 2-dose/placebo and 3-dose/placebo, 1.3 and 1.6, respectively) [6, 16]. Despite high background seroconversion, efficacy against severe rotavirus gastroenteritis in Malawi was 49.2% and 49.7% to 1 year of age for 2 and 3 doses, respectively [6].

In our study, infants with MDNA in the highest quarter tended to have lower seroconversion compared to infants with MDNA in the lowest quarter. However, the difference did not reach statistical significance in the 6/10 arm with the 14-week result, where one would have expected the maximum impact

Downloaded from http://jid.oxfordjournals.org/ at Aga Khan University on January 2, 2015

of MDNA (unless MDNA in even our lowest quarter reduces response at both 6 and 10 weeks). Although based on small numbers of infants and with the contribution of background (wild-type) seroconversion unknown, seroconversion reached 44.7% and 59.1% (overall GMT 38.9 U/mL and 34.8 U/mL for these subsets) in the 6/10/14 and 10/14 arms, respectively, among infants with MDNA in the lowest quarter. High MDNA levels have often been considered one of the important reasons for lack of rotavirus vaccine take in trials of the currently available vaccines; however, the mechanism of interference is not well understood, and data has not been previously published on their impact with RV1 or RV5. Further, with assessment of immune response only after ≥2 doses in a series or with infants receiving doses at a range of ages, it is not possible to discern the true effect of maternal antibody vs some other age-related factor [17]. Comparisons of maternal antibodies in women of child-bearing age from various populations and even in relation to timing of peak rotavirus circulation could help inform the extent to which maternal antibodies are responsible for the lower immune response seen for the current rotavirus vaccines. Possible mechanisms to help overcome their presumed negative impact on vaccine effectiveness in early infancy include providing doses at even older ages (by further increasing total number of doses and/or increasing time between doses) or even development of higher-titer vaccines.

In summary, we found that 3 doses of RV1 administered at 6, 10, and 14 weeks, or delayed administration of 2 doses at 10 and 14 weeks, did not substantially improve the antirotavirus serum IgA response as compared to the 2-dose schedule at 6 and 10 weeks, when doses were provided under tight age windows. These data support the current WHO recommendation to administer RV1 in a 2-dose schedule at the first 2 EPI visits. Continued efforts are needed to increase the immunogenicity and effectiveness of rotavirus vaccine in high burden countries of Asia and Africa.

Notes

Acknowledgments. The authors acknowledge all members of the rotavirus vaccine trial team in Pakistan for their hard work in conducting this trial. They acknowledge all the families who participated in this trial.

Financial support. This work was supported by PATH through funding from the Global Alliance for Vaccines and Immunization (GAVI). The findings and conclusions in this report are those of the author(s) and do not necessarily represent the official position of GAVI, PATH, or the Centers for Disease Control and Prevention.

Potential conflicts of interest. All authors: No potential conflicts of interest.

All authors have submitted the ICMJE Form for Disclosure of Potential Conflicts of Interest. Conflicts that the editors consider relevant to the content of the manuscript have been disclosed.

References

1. Tate JE, Burton AH, Boschi-Pinto C, Steele AD, Duque J, Parashar UD. 2008 estimate of worldwide rotavirus-associated mortality in children younger than 5 years before the introduction of universal rotavirus vaccination programmes: a systematic review and meta-analysis. Lancet Infect Dis **2012**; 12:136–41.
2. Kotloff KL, Nataro JP, Blackwelder WC, et al. Burden and aetiology of diarrhoeal disease in infants and young children in developing countries (the Global Enteric Multicenter Study, GEMS): a prospective, case-control study. Lancet **2013**; 382:209–22.
3. Ruiz-Palacios GM, Perez-Schael I, Velazquez FR, et al. Safety and efficacy of an attenuated vaccine against severe rotavirus gastroenteritis. N Engl J Med **2006**; 354:11–22.
4. Vesikari T, Matson DO, Dennehy P, et al. Safety and efficacy of a pentavalent human-bovine (WC3) reassortant rotavirus vaccine. N Engl J Med **2006**; 354:23–33.
5. Patel MM, Parashar UD. Assessing the effectiveness and public health impact of rotavirus vaccines after introduction in immunization programs. J Infect Dis **2009**; 200 (suppl 1):S291–9.
6. Madhi SA, Cunliffe NA, Steele D, et al. Effect of human rotavirus vaccine on severe diarrhea in African infants. N Engl J Med **2010**; 362: 289–98.
7. Lepage P, Vergison A. Impact of rotavirus vaccines on rotavirus disease. Expert Rev Anti Infect Ther **2012**; 10:547–61.
8. Patel MM, Parashar UD, Santosham M, Richardson V. The rotavirus experience in Mexico: discovery to control. Clin Infect Dis **2013**; 56:548–51.
9. Zaman K, Dang DA, Victor JC, et al. Efficacy of pentavalent rotavirus vaccine against severe rotavirus gastroenteritis in infants in developing countries in Asia: a randomised, double-blind, placebo-controlled trial. Lancet **2010**; 376:615–23.
10. Steele AD, Reynders J, Scholtz F, et al. Comparison of 2 different regimens for reactogenicity, safety, and immunogenicity of the live attenuated oral rotavirus vaccine RIX4414 coadministered with oral polio vaccine in South African infants. J Infect Dis **2010**; 202(supp l):S93–100.
11. The Treatment of diarrhoea: a manual for physicians and other senior health workers. Geneva: World Health Organization, **2005**:44.
12. Bernstein DI, Smith VE, Sherwood JR, et al. Safety and immunogenicity of live, attenuated human rotavirus vaccine 89–12. Vaccine **1998**; 16:381–7.
13. Knowlton DR, Spector DM, Ward RL. Development of an improved method for measuring neutralizing antibody to rotavirus. J Virol Methods **1991**; 33:127–34.
14. Zaman K, Sack DA, Yunus M, et al. Successful co-administration of a human rotavirus and oral poliovirus vaccines in Bangladeshi infants in a 2-dose schedule at 12 and 16 weeks of age. Vaccine **2009**; 27: 1333–9.
15. Madhi SA, Kirsten M, Louw C, et al. Efficacy and immunogenicity of two or three dose rotavirus-vaccine regimen in South African children over two consecutive rotavirus-seasons: a randomized, double-blind, placebo-controlled trial. Vaccine **2012**; 30(suppl 1):A44–51.
16. Cunliffe NA, Witte D, Ngwira BM, et al. Efficacy of human rotavirus vaccine against severe gastroenteritis in Malawian children in the first two years of life: a randomized, double-blind, placebo controlled trial. Vaccine **2012**; 30(suppl 1):A36–43.
17. Ward RL, Knowlton DR, Zito ET, Davidson BL, Rappaport R, Mack ME. Serologic correlates of immunity in a tetravalent reassortant rotavirus vaccine trial. US Rotavirus Vaccine Efficacy Group. J Infect Dis **1997**; 176:570–7.

Downloaded from http://jid.oxfordjournals.org/ at Aga Khan University on January 2, 2015
